# An adaptive voxel-wise iterative satellite interference localization method based on a double-layer UCA antenna

**DOI:** 10.1371/journal.pone.0360855

**Published:** 2026-10-21

**Authors:** Han Li, Jinjie Yao, Muhua Li, Xu Zhang, Kexin Wang, Hao Li, Congmin Hou

**Affiliations:** Shanxi Key Laboratory of Signal Capturing & Processing, School of Information and Communication Engineering, North University of China, Taiyuan, Shanxi, China; SUNY Upstate Medical University Hospital, UNITED STATES OF AMERICA

## Abstract

Aiming to balance localization accuracy, computational complexity, and robustness under low-SNR conditions in three-dimensional interference source localization, this paper proposes an efficient method combining frequency-domain covariance matrix reconstruction (FDCMR) and adaptive voxel iteration. First, the proposed method employs FDCMR-based direction-of-arrival (DOA) estimation for multi-station coarse localization and uses the intersection of direction cones to substantially compress the global search region. An adaptive voxel iteration with exponentially decreasing resolution is then performed within the compressed region for position refinement. Complexity analysis shows that the proposed method replaces exhaustive direct position determination (DPD) evaluation over the global search region with a bounded number of evaluations in a local adaptive voxel region, thereby substantially reducing the spatial-search complexity. Under the considered 2000 m × 2000 m × 2000 m search region with a 5 m spatial resolution, the number of DPD objective-function evaluations is reduced by approximately five orders of magnitude compared with conventional global DPD, while a localization accuracy of about 5 m is maintained at an SNR of 20 dB. At an SNR of −15 dB, the localization error is reduced by approximately 70% compared with the fixed-grid DPD method. These results demonstrate that the proposed method provides an effective balance between localization accuracy, computational efficiency, and low-SNR robustness.

## 1 Introduction

Global Navigation Satellite System (GNSS) technology is widely used in civilian applications because of its high positioning accuracy, wide-area coverage, and flexibility [1]. However, GNSS signals have very low received power, typically about −160 dBm [2]. This makes GNSS receivers, especially those used in uncrewed aerial vehicles (UAVs) and air transportation, highly susceptible to radio-frequency interference. Such interference, whether unintentional or malicious, can reduce the signal-to-noise ratio (SNR), cause loss of lock in tracking loops, and even lead to service interruptions [3,4]. Reliable navigation is important for maintaining UAV control under GPS jamming [5]. Coordinated interception strategies are also required to address threats from multiple rogue drones [6].

In recent years, the traditional method for localizing satellite-navigation interference sources has relied heavily on the two-step localization method. First, each distributed receiver independently estimates intermediate parameters related to the target position, such as time of arrival (TOA), time difference of arrival (TDOA), angle of arrival (AOA), or frequency difference of arrival (FDOA). Then, based on these estimates, geometric or algebraic methods are used to determine the spatial coordinates of the interference source [7–9].

These methods have clear limitations. Although TDOA and FDOA avoid the direct estimation of absolute propagation time and carrier frequency, they still require adequate time-frequency synchronization among the receiving stations. Their localization performance may also be affected by receiver-position errors and additional motion-related measurement errors [10]. Joint processing of parameters such as AOA, TDOA, and FDOA may further increase the computational burden, especially when nonlinear equations or high-dimensional optimization problems are involved. To address these challenges, several improved localization strategies have been proposed.

Ma et al. [11] proposed a low-complexity TDOA/FDOA localization scheme positioned between the traditional two-step method and the direct position determination (DPD) method. This approach significantly reduces computational complexity while maintaining accuracy close to that of DPD, thereby providing a favorable trade-off for real-time localization applications. To improve localization accuracy under different synchronization conditions, Li et al. [12] introduced phase difference of arrival (PDOA) into the TDOA/FDOA framework. By combining time-, frequency-, and phase-difference information, their method improves localization performance under different error conditions. In addition, artificial intelligence provides new solutions for localization problems. Zhang et al. [13] proposed a TDOA localization algorithm based on a neural-network ensemble. The neural networks directly learn the nonlinear relationship between measurement features and source positions, improving the adaptability of data-driven localization in non-line-of-sight environments.

Although these improvement strategies enhance two-step methods to some extent, fundamental limitations remain from the perspective of estimation theory. At each receiving station, parameter estimation is performed independently, ignoring the key constraint that all observed signals originate from the same radiation source. This leads to inefficient use of the received signal information. As modern positioning systems continue to develop in transmission, storage, and computing capability, the traditional computational advantages of two-step methods are gradually diminishing.

To avoid the performance loss caused by independently estimated intermediate parameters, subsequent studies extended DPD to wideband sources [14] and multiple sources observed by moving virtual interpolation arrays [15]. These developments build on the original DPD framework introduced by Weiss [16]. DPD eliminates the parameterized intermediate estimation step and directly processes raw or preprocessed baseband signals. The source position is obtained by constructing and optimizing a cost function in which the source position is the unknown variable.

DPD has favorable theoretical statistical performance, but several practical challenges remain. First, an accurate signal model is required because the location information must be directly associated with specific signal features [17,18]. Second, DPD usually requires a grid search over the candidate region. Its cost function is often non-convex and may contain multiple local extrema [19]. If the grid is too fine, the computational burden becomes excessive; if the grid is too coarse, large localization errors may occur.

To reduce the computational complexity of DPD, various improved methods have been proposed. Chen et al. [20] developed a neural-network-based DPD method that avoids computationally expensive spectral peak searches and iterative optimization. Xia and Liu [21] proposed a distributed adaptive DPD method that reduces communication and computational burdens by distributing signal processing among neighboring sensor nodes. Hao and Wan [22] developed an importance-sampling-based maximum-likelihood DPD method that decomposes the multidimensional optimization problem into several lower-dimensional subproblems. Zhou et al. [23] combined an iterative adaptive approach with DPD for the direct localization of multiple coherent sources.

Although three-dimensional DPD and array-orientation optimization have been investigated [24], much of the existing DPD literature still focuses on planar or simplified localization geometries. Moreover, array-model errors may cause additional performance degradation in practical DPD systems [25]. For UAV and low-altitude-aircraft applications, horizontal-plane information alone is insufficient because the source position varies in both the horizontal and vertical dimensions.

Three-dimensional source localization requires the estimation of azimuth, elevation, and range. Recent studies have investigated joint azimuth, elevation, and delay estimation for single-base-station three-dimensional localization [26], as well as three-dimensional emitter geolocation using azimuth and elevation measurements from a single aerial platform [27]. The uniform circular array (UCA) is widely used because of its capability for two-dimensional DOA estimation, omnidirectional azimuth coverage, and potential for extracting elevation information [28]. Jung et al. [29] proposed a closed-form three-dimensional single-source localization algorithm using a UCA. Zuo et al. [30] developed an analytical algorithm that exploits the central symmetry and periodicity of the UCA in the Fourier domain. However, these methods mainly focus on angular or parameter estimation rather than direct multi-station localization.

To reduce the computational complexity of conventional three-dimensional multiple signal classification (MUSIC) algorithms, Wu et al. [31] proposed a reduced-dimension MUSIC algorithm based on a sparse symmetric cross array. This algorithm converts an exhaustive three-dimensional search into three one-dimensional searches, substantially reducing computational cost. However, it is mainly designed for near-field source localization and may be less suitable for far-field interference sources. Ni et al. [32] proposed a fast DPD method based on particle swarm optimization to reduce the burden of exhaustive search. Nevertheless, intelligent optimization methods may still exhibit sensitivity to initialization and limited stability under low-SNR conditions.

To address these challenges, this paper proposes an adaptive voxel iterative method for three-dimensional satellite-interference-source localization based on a double-layer UCA. First, the double-layer UCA configuration uses the vertical phase difference between the two array layers to improve pitch-angle discrimination. Second, frequency-domain covariance matrix reconstruction (FDCMR)-based DOA estimation [33] is combined with direction-cone intersection to compress the original global search space into a compact candidate region before DPD evaluation. Third, an adaptive voxel iteration strategy refines the source position within the compressed region, thereby substantially reducing the number of DPD objective-function evaluations while maintaining localization accuracy and low-SNR robustness. The theoretical analysis and simulation results demonstrate that the proposed method provides a practical accuracy-complexity trade-off for three-dimensional interference-source localization.

## 2 Theoretical analysis

Accurate three-dimensional localization of interference sources is important for both theoretical analysis and practical applications. Compared with two-dimensional localization, it provides more complete spatial information for subsequent interference suppression and spectrum management.

### 2.1 Double-layer UCA configuration and signal model

Although a conventional single-layer uniform circular array (UCA) performs well in azimuth estimation, its pitch-angle estimation accuracy is limited. To improve pitch-angle resolution, a double-layer UCA is adopted. As shown in Fig 1, the configuration consists of two vertically stacked, co-centered UCAs, namely UCA1 and UCA2. The upper UCA contains $N_{\text{u}}$ elements with radius $R_{\text{u}}$, and the m -th upper-layer element has the angular position$\phi_{m}=\frac{2\pi (m-1)}{N_{\text{u}}}$, The lower UCA contains $N_{\text{d}}$ elements with radius $R_{\text{d}}$, and the n -th lower-layer element has the angular position $\phi_{n}=\frac{2\pi (n-1)}{N_{\text{d}}}$. The inter-layer spacing is denoted by h, and the total number of array elements is $M=N_{\text{u}}+N_{\text{d}}$.

**Fig 1:**
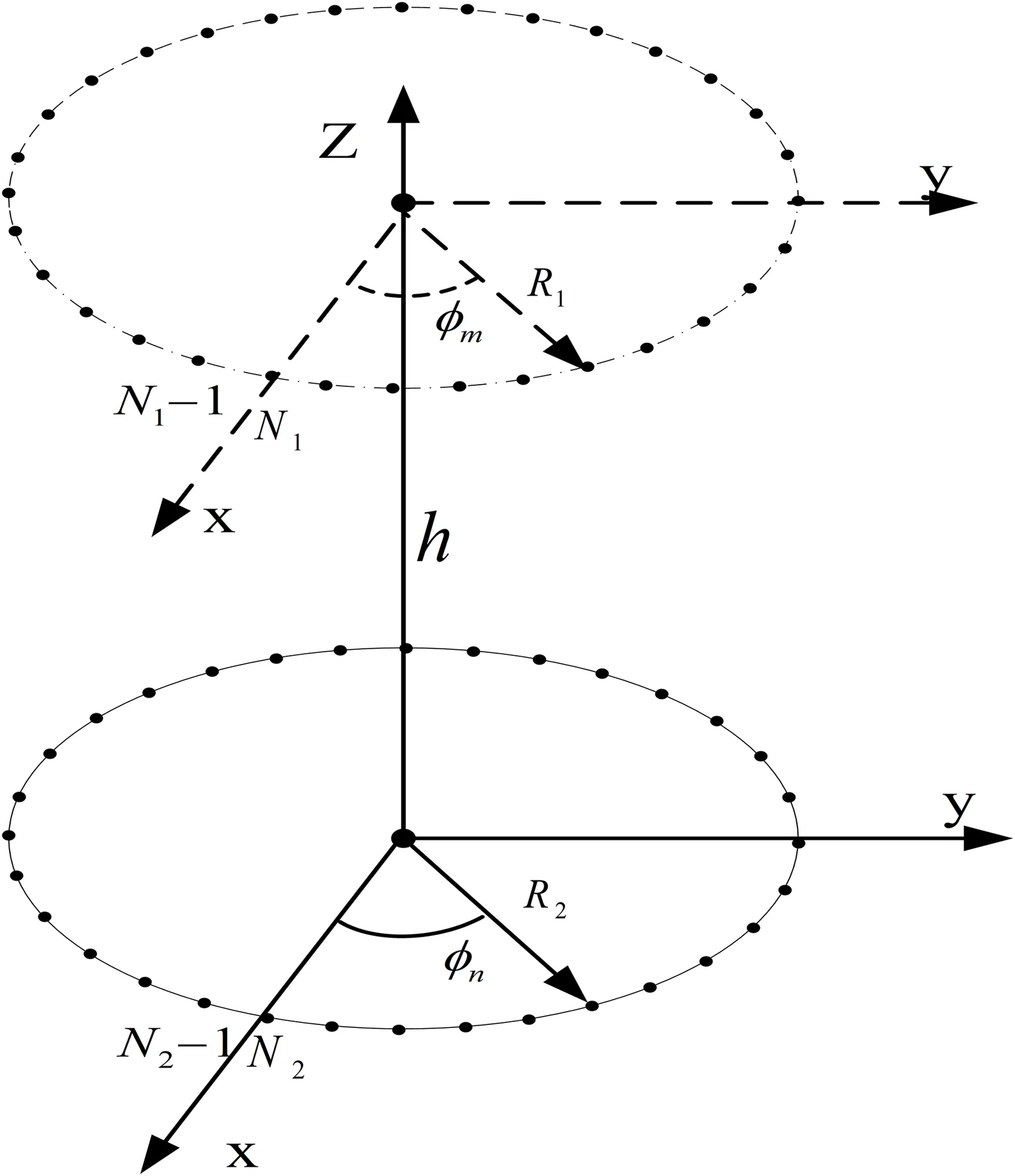
Schematic diagram of the antenna structure. The configuration illustrates the two vertically stacked, co-centered UCAs (UCA1 and UCA2) with key geometric parameters.

Consider a plane wave arriving from azimuth angle θ and pitch angle ϕ, where θ denotes the azimuth angle and ϕ denotes the pitch angle. The wave vector is defined as


$$\begin{array}{c} 𝐤=\frac{2\pi }{\lambda }[cos\phi cos\theta ,cos\phi sin\theta ,sin\phi {]}^{T}, \end{array}$$ (1)

where λ denotes the signal wavelength.

For an antenna element i with position vector ${𝐫}_{i}$ relative to the array center, the phase delay $\Delta \psi_{i}\;$ is given by


$$\begin{array}{c} \Delta \psi_{i}\;=\;{𝐤}^{T}{𝐫}_{i}, \end{array}$$ (2)

where i is the antenna-element index, ${𝐫}_{i}$ is the position vector of the i -th antenna element relative to the array center, and $\Delta \psi_{i}\;$ is the corresponding phase delay.

For the proposed double-layer UCA, the steering vector $𝐚\;\left(\theta ,\phi \right)\in {\mathbb{C}}^{M\times 1}$ is formed by cascading the steering vectors of the upper and lower UCA layers. To make the notation clearer, the steering vectors of the upper and lower layers are denoted as ${𝐚}_{\text{u}}\left(\theta ,\varphi \right)$ and ${𝐚}_{\text{d}}\left(\theta ,\varphi \right)$, respectively.


$$\begin{array}{c} \;𝐚\left(\theta ,\varphi \right)=\left[\begin{array}{c} {𝐚}_{\text{u}}\left(\theta ,\varphi \right)\\ {𝐚}_{\text{d}}\left(\theta ,\varphi \right) \end{array}\right]\in {\mathbb{C}}^{M\times 1}. \end{array}$$ (3)

Here, ${𝐚}_{u}\left(\theta ,\phi \right)\in {\mathbb{C}}^{N_{u}\times 1}$ and ${𝐚}_{d}\left(\theta ,\phi \right)\in {\mathbb{C}}^{N_{d}\times 1}$ represent the steering vectors of the upper and lower UCAs, respectively.

The m -th element of the upper-UCA steering vector contains both horizontal and vertical phase-delay terms, where $m=1,2,...,N_{\text{u}}\;$:


$$\begin{array}{c} {\left[{𝐚}_{\text{u}}\left(\theta ,\varphi \right)\right]}_{m}=exp\left\{\text{j}\frac{2\pi }{\lambda }\left[R_{\text{u }}cos\varphi cos(\theta -\phi_{\text{u},m})+hsin\varphi \right]\right\}. \end{array}$$ (4)

The n -th element of the lower-UCA steering vector contains only the horizontal phase-delay term, where $n=1,2,...,N_{\text{d}}\;$:


$$\begin{array}{c} {\left[{𝐚}_{\text{d}}\left(\theta ,\varphi \right)\right]}_{n}=exp\left\{\text{j}\frac{2\pi }{\lambda }R_{\text{d }}cos\varphi cos(\theta -\phi_{\text{d},n})\right\}, \end{array}$$ (5)

where $\phi_{m\;}=\frac{2\pi (m-1)\;}{N_{\text{u}}}$ and $\phi_{n\;}=\frac{2\pi (n-1)\;}{N_{\text{d}}}$ are the angular positions of the m -th upper-layer element and the n -th lower-layer element, respectively. The term $\frac{2\pi }{\lambda }\;h\;sin\phi$ represents the vertical phase difference introduced by the inter-layer spacing h, which improves the pitch-angle distinguishability of the double-layer UCA.

Consider a static three-dimensional single-interference-source localization scenario, as shown in Fig 2. The true position of the interference source is denoted as ${𝐩}_{s}=[x_{s},y_{s},z_{s}{]}^{T}$. There are L static receivers, and the position of the l -th receiver is denoted as ${𝐩}_{l}=[x_{l},y_{l},z_{l}{]}^{T}$, where l=1,2,...,L. Each receiver is equipped with a double-layer UCA containing M array elements.

**Fig 2:**
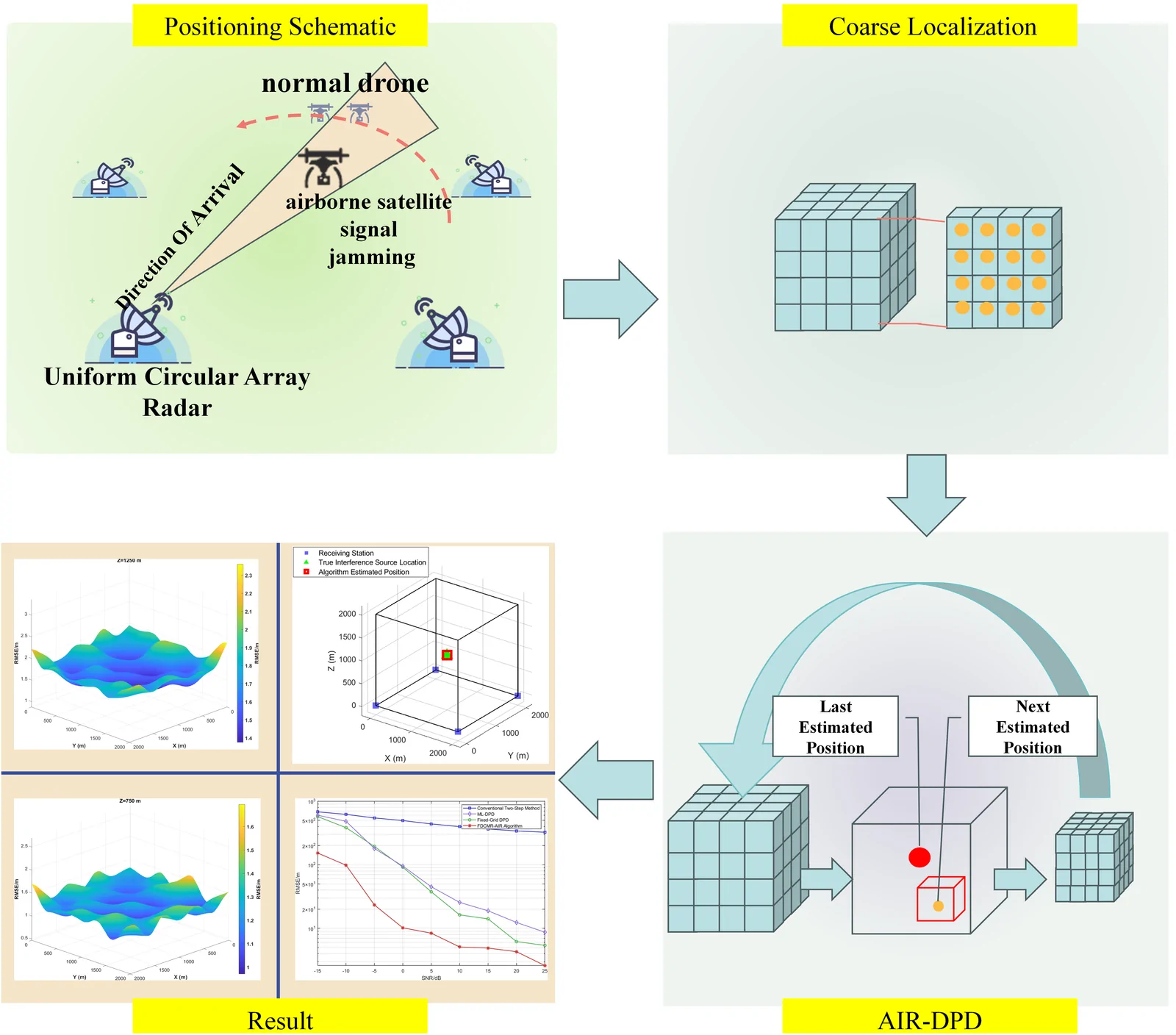
Schematic diagram of algorithm structure. The flowchart outlines the three-stage process: FDCMR-based DOA estimation, conical intersection for coarse localization, and adaptive voxel iteration for refinement.

Under the above assumptions, if the interference source transmits s(t), the signal received by the l -th receiver is modeled as follows.


$$\begin{array}{c} {𝐱}_{l}\left(t\right)=\alpha_{l}\cdot {𝐚}_{l}\left(\theta_{l},\phi_{l}\right)\cdot s(t-\tau_{l})+{𝐧}_{l}\left(t\right), \end{array}$$ (6)

where ${𝐱}_{l}\left(t\right)\in {\mathbb{C}}^{M\times 1}$ is the received signal vector of the l -th receiver, $\alpha_{l}$ is the complex path gain, ${𝐚}_{l}\left(\theta_{l},\phi_{l}\right)\in {\mathbb{C}}^{M\times 1}$ is the double-layer UCA steering vector at the l -th receiver, $s(t-\tau_{l})$ is the delayed transmitted signal, $\tau_{l}$ is the propagation delay, and ${𝐧}_{l}\left(t\right)\in {\mathbb{C}}^{M\times 1}$ is the additive white Gaussian noise (AWGN) vector..

The location information of the interference source is contained in both ${𝐚}_{l}\left(\theta_{l},\phi_{l}\right)$ and $\tau_{l}$. The azimuth angle $\theta_{l}$ and pitch angle $\phi_{l}$ are determined by the relative geometry between ${𝐩}_{s}$ and ${𝐩}_{l}$. The propagation delay is calculated as $\tau_{l}=\frac{∥{𝐩}_{s}-{𝐩}_{l}{∥}_{\text{2}}}{c}$ where c is the speed of light.

The received signal is uniformly sampled with sampling interval $T_{s}$, and the discrete-time signal is obtained as


$$\begin{array}{c} {𝐱}_{l}\left[n\right]={𝐱}_{l}\left(t\right)|{\;}_{t=nTs}=\alpha_{l}\cdot {𝐚}_{l}\left(\theta_{l},\phi_{l}\right)\cdot s(nT_{s}-\tau_{l})+{𝐧}_{l}\left[n\right],n=0,1,...,N_{s}-1. \end{array}$$ (7)

Here, n is the time-sample index, $N_{s}$ is the number of time-domain samples, and ${𝐱}_{l}\left[n\right]$ and ${𝐧}_{l}\left[n\right]$ denote the discrete-time received signal vector and noise vector of the l -th receiver, respectively.

After applying the discrete Fourier transform, the frequency-domain received signal is expressed as


$$\begin{array}{c} {𝐗}_{l}\left[k\right]=\text{FFT}\left\{{𝐱}_{l}\left(n\right)\right\}=\alpha_{l}\cdot {𝐚}_{l}\left(\theta_{l},\phi_{l}\right)\cdot S\left[k\right]\cdot e^{-j2\pi kf_{k}\tau_{l}}+{𝐍}_{l}\left[k\right], \end{array}$$ (8)

where ${𝐗}_{l}\left[k\right]\in {\mathbb{C}}^{\left(M\times 1\right)}$ is the frequency-domain received vector of the l -th receiver at the k -th frequency bin, S[k] is the DFT coefficient of the transmitted signal, ${𝐍}_{l}\left[k\right]$ is the DFT coefficient of the noise vector, and $f_{k}$ is the frequency corresponding to the k -th frequency bin.

The joint received-signal vector from all L base stations is defined as follows:


$$\begin{array}{c} 𝐱\left(t\right)={\left[\begin{array}{c} {𝐱}_{\text{1}}^{T}\left(t\right)\\ {𝐱}_{\text{2}}^{T}\left(t\right)\\ \ldots \\ {𝐱}_{L}^{T}\left(t\right) \end{array}\right]}^{T}\in {\mathbb{C}}^{LM\times 1}. \end{array}$$ (9)

Accordingly, the corresponding joint steering vector for all L base stations is defined as follows:


$$\begin{array}{c} A\left(𝐩\right)=\left[\begin{array}{c} \alpha_{\text{1}}{𝐚}_{\text{1}}\left(\theta_{\text{1}},\phi_{\text{1}}\right)e^{-j2\pi f_{k}\tau_{\text{1}}}\\ \alpha_{\text{2}}{𝐚}_{\text{2}}\left(\theta_{\text{2}},\phi_{\text{2}}\right)e^{-j2\pi f_{k}\tau_{\text{2}}}\\ \vdots \\ \alpha_{L}{𝐚}_{L}\left(\theta_{L},\phi_{L}\right)e^{-j2\pi f_{k}\tau_{L}} \end{array}\right]\in {\mathbb{C}}^{LM\times 1}, \end{array}$$ (10)

where $A\left(𝐩\right)\in {\mathbb{C}}^{LM\times 1}$ is the joint steering vector of all base stations at candidate position 𝐩, and each block $\alpha_{\text{1}}{𝐚}_{\text{1}}\left(\theta_{\text{1}},\phi_{\text{1}}\right)e^{-j2\pi f_{k}\tau_{\text{1}}}$ represents the delay-compensated steering response of the l -th base station.

### 2.2 DOA estimation

According to the received signal model established in Section 2.1, the received signal of the double UCA at the base station l is as follows:


$$\begin{array}{c} {𝐱}_{l}\left(t\right)=\alpha_{l}\cdot {𝐚}_{l}\left(\theta_{l},\phi_{l}\right)\cdot s(t-\tau_{l})+{𝐧}_{l}\left(t\right). \end{array}$$ (11)

The received signal of each antenna element is uniformly sampled with sampling interval $T_{s}$. The frequency-domain representation is then obtained for DOA estimation.

#### 2.2.1 Frequency domain transformation and target frequency extraction.

First, a $N_{s}$ -point Fast Fourier transform (FFT) is applied to the received signal from each antenna to convert the signal to the frequency domain:


$$\begin{array}{c} {𝐗}_{l}^{\left(n\right)}\;\left(k\right)=\sum_{m=0}^{N_{s}-1}{𝐱}_{l}^{\left(n\right)}\left[m\right]e^{-j\frac{2\pi }{N_{s}}\;mk},\;\}n=1,2,\ldots ,M\;\}\;\;\;\;\;, \end{array}$$ (12)

where ${𝐗}_{l}^{\left(n\right)}\;\left(k\right)$ denotes the frequency-domain signal of the n -th antenna element at the l -th receiver and the k -th frequency bin, ${𝐱}_{l}^{\left(n\right)}\left[m\right]$ denotes the m -th time-domain sample of the n -th antenna element, m is the time-sample index, k is the frequency-bin index, $N_{s}$ is the number of FFT points, and M is the total number of array elements in the double-layer UCA.

After power spectrum analysis, $N_{f}$ significant spectral peaks are selected, and the frequency-domain data at the corresponding frequency points are extracted to form the observation matrix


$$\begin{array}{c} {𝐗}_{F,l}\;=[{𝐗}_{l}\;(f_{l}\;),{𝐗}_{l}(f_{\text{3}}\;),\ldots ,{𝐗}_{l}\;(f_{N_{f}}\;){]}^{T}\in {\mathbb{C}}^{M\times N_{f}}. \end{array}$$ (13)

Here, each column corresponds to the received frequency-domain snapshot at one selected frequency point.

#### 2.2.2 Reconstruction of the covariance matrix in the frequency domain.

For the l -th receiver, the frequency-domain covariance matrix is reconstructed as


$$\begin{array}{c} {𝐑}_{F,l}\;=\frac{\text{1}}{N_{f}}\;{𝐗}_{F,l}{𝐗}_{F,l}^{H}, \end{array}$$ (14)

where ${𝐑}_{F,l}\;\in {\mathbb{C}}^{\left(M\times M\right)}$ is the reconstructed frequency-domain covariance matrix of the l -th receiver, ${𝐗}_{F,l}\;\in {\mathbb{C}}^{\left(M\times N_{f}\right)}$ is the frequency-domain observation matrix, $N_{f}$ is the number of selected frequency-domain samples and $(\cdot {)}^{H}$ denotes the Hermitian transpose..

For the double-layer UCA, the steering vector ${𝐚}_{l}\left(\theta ,\phi \right)$ contains both horizontal and vertical phase information. In particular, the vertical phase difference introduced by the inter-layer spacing h enhances the distinguishability of the pitch angle ϕ.

#### 2.2.3 Frequency domain noise subspace extraction and FDCMR spectrum estimation.

Eigenvalue decomposition is performed on the reconstructed covariance matrix $R_{F,l}\;$ as follows:


$$\begin{array}{c} {𝐑}_{F,l}\;={𝐔}_{F,l\;}\Sigma_{F,l}\;{𝐔}_{F,l}^{H}, \end{array}$$ (15)

where ${𝐔}_{F,l\;}=\left[{𝐔}_{F,S,l}\;,{𝐔}_{F,N,l\;}\right]$ is the eigenvector matrix of ${𝐑}_{F,l}$, $\Sigma_{F,l}\;$ is the diagonal eigenvalue matrix, ${𝐔}_{F,S,l}\;$ denotes the frequency-domain signal subspace, and ${𝐔}_{F,N,l\;}$ denotes the frequency-domain noise subspace.

By leveraging the orthogonality between the frequency-domain noise subspace and the steering vector, the FDCMR spatial spectral function is constructed as


$$\begin{array}{c} {𝐏}_{l}\;\left(\theta ,\phi \right)=\frac{\text{1}}{{𝐚}_{l}^{H}\;\left(\theta ,\phi \right){𝐔}_{F,N,l\;}\;{𝐔}_{{\;}_{F,N,l\;}}^{H}\;{𝐚}_{l}\;\left(\theta ,\phi \right)}, \end{array}$$ (16)

where ${𝐏}_{l}\;\left(\theta ,\phi \right)$ denotes the FDCMR spatial spectrum value of the l -th receiver at the candidate direction (θ,ϕ).

#### 2.2.4 Coarse positioning based on conical intersection of multiple base stations.

In three-dimensional space, however, each single-station DOA estimate $\{\left({\hat{\theta }}_{l},{\hat{\phi }}_{l}\right){\}}_{l=1}^{L}$ defines a direction cone whose vertex is located at the antenna phase center of the corresponding station. The central axis of the cone points in the estimated direction, and its opening angle is determined by the DOA estimation uncertainty.

The unit direction vector corresponding to the DOA estimate of the l -th base station is defined consistently with the wave-vector convention in Section 2.1 as ${\hat{𝐮}}_{l}=[cos{\hat{\phi }}_{l}cos{\hat{\theta }}_{l},cos{\hat{\phi }}_{l}sin{\hat{\theta }}_{l},sin{\hat{\phi }}_{l}{]}^{T}$.

For the l -th receiving station, each candidate source position $𝐩=[x,y,z{]}^{T}$ inside the direction cone satisfies


$$\begin{array}{c} \frac{(𝐩-{𝐩}_{l}{)}^{T}{\hat{𝐮}}_{l}}{∥𝐩-{𝐩}_{l}{∥}_{\text{2}}}\geq cos\left(\delta_{\phi }\right), \end{array}$$ (17)

where ${𝐩}_{{\;}_{l}}$ represents the three-dimensional coordinates of the l -th receiving station, and $\delta_{\phi }$ is the preset angular error tolerance. This inequality represents the cone constraint induced by the DOA estimation uncertainty.

The actual location of the interference source should lie within the direction cones of multiple base stations. Therefore, coarse positioning is transformed into identifying the candidate voxels that satisfy most of the direction-cone constraints. To balance computational efficiency and localization accuracy, the following strategy is adopted.

Direction-cone generation: Based on the DOA estimation values $\left({\hat{\theta }}_{l},{\hat{\phi }}_{l}\right)$ of each base station and the preset angular error tolerance $\delta_{\phi }$, a circular cone with opening angle $2\delta_{\phi }$ and central axis ${\hat{𝐮}}_{l}$ is used to represent the uncertainty region of the DOA estimation.

Intersection-voxel identification: The three-dimensional search space is divided into grid voxels. For each voxel, the consistency between its direction and the direction cones of different base stations is evaluated. Voxels covered by most of the direction cones are marked as candidate voxels.

Search-region definition: The selected candidate voxels jointly form a compact three-dimensional candidate region V, which is much smaller than the original global search space and is used for subsequent adaptive refinement.

Initial-position determination: After the candidate region V is obtained, the initial position ${𝐩}_{\text{0}}$ is selected as the geometric center of all candidate voxels in V.

### 2.3 Adaptive Voxel Iteration

After FDCMR-DOA estimation at multiple receivers and direction-cone intersection, the original search space is compressed into a much smaller three-dimensional candidate region. When the DOA estimation errors remain within the preset angular tolerance, this region is expected to contain the true source position. However, exhaustive grid search within the compressed region can still be computationally expensive, whereas random optimization algorithms may exhibit unstable convergence. Therefore, adaptive voxel iteration is employed to refine the source-position estimate within this region.

The adaptive voxel iteration provides a deterministic refinement process with controllable computational cost. Through systematic grid contraction and exponentially decreasing resolution, it progressively refines the source-position estimate within a limited number of iterations.

#### 2.3.1 Problem modeling.

The localization problem can be formulated as an optimization problem, with the objective of finding a candidate source position $𝐩=[x,y,z{]}^{T}$ such that the spatial consistency of the compensated received signals from different base stations is maximized. The fitness function is based on the DPD principle and fully utilizes the frequency-domain signals received by the double-layer UCA.

For L base stations, the frequency-domain received signal of the l -th base station is denoted as ${𝐗}_{l}\;\left(f\right)$. For a candidate source position $𝐩=[x,y,z{]}^{T}$, after time-delay compensation, phase compensation, and steering-vector matching with the double-layer UCA, the matched filtering output of the l -th base station is denoted as ${𝐮}_{l}\left(𝐩\right)\in {\mathbb{C}}^{N_{f}\times 1}$, where $N_{f}$ is the number of selected frequency-domain samples.

The joint observation matrix is then constructed as


$$\begin{array}{c} 𝐔\left(𝐩\right)=\left[{𝐮}_{\text{1}}\left(𝐩\right),{𝐮}_{\text{2}}\left(𝐩),\ldots ,{𝐮}_{L}(𝐩\right)\right]\in {\mathbb{C}}^{N_{f}\times L}, \end{array}$$ (18)

where the l -th column of 𝐔(𝐩) corresponds to the compensated matched filtering output of the l -th base station. To evaluate the spatial consistency among different base stations, the following Hermitian matrix is defined:


$$\begin{array}{c} 𝐃\left(𝐩\right)={𝐔}^{H}\left(𝐩\right)𝐔\left(𝐩\right)\in {\mathbb{C}}^{L\times L}. \end{array}$$ (19)

The DPD fitness function is then defined as the maximum eigenvalue of 𝐃(𝐩):


$$\begin{array}{c} f\left(𝐩\right)=\lambda_{\text{max}}\left(𝐃\left(𝐩\right)\right)=\lambda_{\text{max}}\left({𝐔}^{H}\left(𝐩\right)𝐔\left(𝐩\right)\right), \end{array}$$ (20)

where $\lambda_{max}(\cdot )$ denotes the maximum eigenvalue of a matrix. Since 𝐃(𝐩) is an L×L Hermitian matrix, f(𝐩) is a scalar value.

The use of the maximum eigenvalue is directly related to the DPD principle. For a candidate position 𝐩, the received signals from all base stations are compensated according to the propagation delay, phase shift, and steering vector corresponding to 𝐩. If 𝐩 is close to the true source position, the compensated matched-filtering outputs from different base stations become highly coherent and share the same source waveform component. In this case, the joint observation matrix 𝐔(𝐩) contains a dominant rank-one signal component, and the Hermitian matrix $𝐃\left(𝐩\right)={𝐔}^{H}\left(𝐩\right)𝐔\left(𝐩\right)\in {\mathbb{C}}^{L\times L}$ has a dominant principal eigenvalue.

Under the AWGN model, the noise components at different base stations are uncorrelated and have no preferred spatial direction. Therefore, the noise contribution is spread over the eigenspectrum, while the coherent signal component corresponding to the correct position is concentrated in the largest eigenvalue. Maximizing $\lambda_{max}\left(𝐃\left(𝐩\right)\right)$ is thus equivalent to searching for the candidate position that produces the strongest coherent signal component after DPD compensation. Therefore, the maximum eigenvalue is selected as the DPD fitness metric in this study, and the optimization objective is to maximize f(𝐩).

#### 2.3.2 Adaptive iterative algorithm flow.

The adaptive voxel iterative method employs a systematic grid contraction strategy to improve convergence stability while significantly enhancing computational efficiency. This method uses the coarse positioning result ${𝐩}_{\text{0}}$ as the initial search center and gradually improves the localization accuracy through multiple iterations. The current optimal position is initialized as ${\hat{𝐩}}^{\left(0\right)}={𝐩}_{\text{0}}$.

The initial grid resolution is set to $d^{\left(0\right)}$, the coverage parameter is i, the maximum number of iterations is $K_{max}$, and the convergence threshold is ε.

At the k -th iteration, the search grid is constructed using the resolution


$$\begin{array}{c} d^{\left(k-1\right)}=\frac{d^{\left(0\right)}}{{\text{2}}^{k-1}},\;\;\}k=1,2,\ldots ,K_{max}. \end{array}$$ (21)

Based on this resolution, a three-dimensional uniform search grid ${𝐆}^{\left(k\right)}$ is constructed with the current optimal position ${\hat{𝐩}}^{\left(k-1\right)}$ as the center.

The candidate coordinate sets are constructed as


$$\begin{array}{c} \begin{array}{c} {𝐗}^{\left(k\right)}=\left\{{\hat{p}}_{x}^{\left(k-1\right)}-i\cdot d^{\left(k-1\right)},{\hat{p}}_{x}^{\left(k-1\right)}-(i-1)\cdot d^{\left(k-1\right)},\ldots ,{\hat{p}}_{x}^{\left(k-1\right)}+i\cdot d^{\left(k-1\right)}\right\},\\ {𝐘}^{\left(k\right)}=\left\{{\hat{p}}_{y}^{\left(k-1\right)}-i\cdot d^{\left(k-1\right)},{\hat{p}}_{y}^{\left(k-1\right)}-(i-1)\cdot d^{\left(k-1\right)},\ldots ,{\hat{p}}_{y}^{\left(k-1\right)}+i\cdot d^{\left(k-1\right)}\right\},\\ {𝐙}^{\left(k\right)}=\left\{{\hat{p}}_{z}^{\left(k-1\right)}-i\cdot d^{\left(k-1\right)},{\hat{p}}_{z}^{\left(k-1\right)}-(i-1)\cdot d^{\left(k-1\right)},\ldots ,{\hat{p}}_{z}^{\left(k-1\right)}+i\cdot d^{\left(k-1\right)}\right\}. \end{array} \end{array}$$ (22)

Here, ${𝐗}^{\left(k\right)}$, ${𝐘}^{\left(k\right)}$, and ${𝐙}^{\left(k\right)}$ denote the candidate coordinate sets along the x-, y-, and z-directions at the k -th iteration. The quantities ${\hat{p}}_{x}^{\left(k-1\right)}$, ${\hat{p}}_{y}^{\left(k-1\right)}$, and ${\hat{p}}_{z}^{\left(k-1\right)}$ are the coordinates of the estimate obtained in the previous iteration, and $d^{\left(k-1\right)}$ is the grid resolution used at the k -th iteration.

There are 2i+1 grid points in each coordinate direction, so the total number of candidate points is ${\left(2i+1\right)}^{\text{3}}$. For each candidate position $𝐩=[x,y,z{]}^{T}\in {𝐗}^{\left(k\right)}\times {𝐘}^{\left(k\right)}\times {𝐙}^{\left(k\right)}$, the DPD fitness function based on the Hermitian evaluation matrix is calculated as follows:


$$\begin{array}{c} f\left(𝐩\right)=\lambda_{max\;}\left(D\left(𝐩\right)\right). \end{array}$$ (23)

Here, D(𝐩) denotes the Hermitian DPD evaluation matrix defined in Section 2.3.1, and f(𝐩) reflects the spatial coherence of all base stations at the candidate position.

The candidate position with the maximum fitness function is selected as the optimal estimate for this iteration:


$$\begin{array}{c} {\hat{𝐩}}^{\left(k\right)}=\underset{𝐩\in G^{\left(k\right)}}{argmax}f\left(𝐩\right). \end{array}$$ (24)

For each candidate location in the grid, its fitness function value f(𝐩) is computed in parallel. By comparing the fitness values of all candidate points, the optimal position ${\hat{𝐩}}^{\left(k\right)}$ for this iteration is determined. The convergence criterion is based on the Euclidean distance between the position estimates obtained in two adjacent iterations. If $∥{\hat{𝐩}}^{\left(k\right)}-{\hat{𝐩}}^{\left(k-1\right)}{∥}_{\text{2}}<\varepsilon$, the convergence criterion is satisfied and the iterative process is terminated early. Otherwise, ${\hat{𝐩}}^{\left(k\right)}$ is used as the search center for the next iteration.

The algorithm terminates when either the convergence criterion is satisfied or the maximum number of iterations $K_{\text{max}}$ is reached. The final source-position estimate is then output as $𝐩={\hat{𝐩}}^{\left(k\right)}$. Through the exponential decrease in grid resolution and the systematic contraction of the search region, the method progressively refines the position estimate while limiting the computational cost.

**Algorithm 1: Voxel-wise Adaptive Iterative Localization**

**1:Input:** FDCMR-DOA estimates from multiple base stations; initial search center ${𝐩}_{\text{0}}$; initial grid resolution $d^{\left(0\right)}$; coverage factor i; maximum iteration count $K_{max}$; convergence threshold ε.

**2:Output:** Precise estimated location $\hat{𝐩}$ of the interference source.

**3: Initialize:** Set k=1 and ${\hat{𝐩}}^{\left(0\right)}={𝐩}_{\text{0}}$.

**Main Loop:**

**4: while** $k\leq K_{max}$ do

5: Update grid resolution: $d^{\left(k-1\right)}=\frac{d^{\left(0\right)}}{{\text{2}}^{k-1}}$

6: Construct the three-dimensional search grid ${𝐆}^{\left(k\right)}$ centered at ${\hat{𝐩}}^{\left(k-1\right)}$ using the grid resolution $d^{\left(k-1\right)}$.

7: for each candidate position $𝐩\in {𝐆}^{\left(k\right)}$ do

8: Calculate fitness function $f\left(𝐩\right)=\lambda_{max\;}\left(D\left(𝐩\right)\right)$

**9: end for**

10: Find optimal point ${\hat{𝐩}}^{\left(k\right)}=\underset{𝐩\in {𝐆}^{\left(k\right)}}{argmax}\;\}f\left(𝐩\right)$

**11: if** ${\|{\hat{𝐩}}^{\left(k\right)}-{\hat{𝐩}}^{\left(k-1\right)}\|}_{\text{2}}<\varepsilon \;or\;\}k=K_{max}$ then

**12: break**

13: **end if**

14: Update center: $𝐩={\hat{𝐩}}^{\left(k\right)}$

15: k=k+1

16: **end while**

17: **Output** final estimated location $\hat{𝐩}={\hat{𝐩}}^{\left(k\right)}$

**Proposition 1. Convergence of the adaptive voxel iteration** Consider the idealized position sequence generated by continuing the adaptive refinement beyond the finite iteration cap. Assume that the search grid ${𝐆}^{\left(k\right)}$ is centered at ${𝐩}^{\left(k-1\right)}$, contains the center point, and has a maximum displacement of $id^{\left(k-1\right)}$ in each coordinate direction, where $d^{\left(k-1\right)}=\frac{d^{\left(0\right)}}{{\text{2}}^{k-1}}.$

Assume further that the DPD fitness function is finite over the bounded candidate region. Then, the sequence of fitness values is nondecreasing and convergent, and the position sequence $\left\{{𝐩}^{\left(k\right)}\right\}$ is a Cauchy sequence that converges to a finite position estimate.

**Proof.** Because the center point ${𝐩}^{\left(k-1\right)}$ is included in ${𝐆}^{\left(k\right)}$, and ${\hat{𝐩}}^{\left(k\right)}$ is selected as the candidate with the maximum fitness value, we have $f\;\left({\hat{𝐩}}^{\left(k\right)}\right)\geq f\;\left({\hat{𝐩}}^{\left(k-1\right)}\right).$

Moreover, because $𝐃\left(𝐩\right)={𝐔}^{H}\left(𝐩\right)𝐔\left(𝐩\right)$ is positive semidefinite,


$$\begin{array}{c} 0\leq f\left(𝐩\right)=\lambda_{max}\;\left(𝐃\left(𝐩\right)\right)\leq tr\;\left(𝐃\left(𝐩\right)\right)={\|𝐔\left(𝐩\right)\|}_{F}^{\text{2}}<\infty . \end{array}$$ (25)

Therefore, $f\;\left({\hat{𝐩}}^{\left(k\right)}\right)$ is a nondecreasing sequence bounded from above and hence converges.

Because the maximum displacement in each coordinate direction is $id^{\left(k-1\right)}$, the distance between two adjacent position estimates satisfies


$$\begin{array}{c} {\|{\hat{𝐩}}^{\left(k\right)}-{\hat{𝐩}}^{\left(k-1\right)}\|}_{\text{2}}\leq \sqrt{\text{3}}\;\}i\;\}d^{\left(k-1\right)}=\frac{\sqrt{\text{3}}\;}{i}\;\}d^{\left(0\right)}\}{\text{2}}^{k-1}. \end{array}$$ (26)

For any two iteration indices m>n, the triangle inequality gives


$$\begin{array}{c} {\|{\hat{𝐩}}^{\left(m\right)}-{\hat{𝐩}}^{\left(n\right)}\|}_{\text{2}}\leq \sum_{k=n+1}^{m}{\|{\hat{𝐩}}^{\left(k\right)}-{\hat{𝐩}}^{\left(k-1\right)}\|}_{\text{2}}\leq \sqrt{\text{3}}\;\}i\;\}d^{\left(0\right)}\sum_{k=n+1}^{m}{\text{2}}^{-(k-1)}\leq \sqrt{\text{3}}\;\}i\;\}d^{\left(0\right)}{\text{2}}^{1-n}. \end{array}$$ (27)

The upper bound approaches zero as n→∞. Therefore, $\left\{{\hat{𝐩}}^{\left(k\right)}\right\}$ is a Cauchy sequence. Because ${\mathbb{R}}^{\text{3}}$ is complete, the sequence converges to a finite position estimate ${\hat{𝐩}}^{\left(\infty \right)}$. Furthermore, its remaining displacement satisfies


$$\begin{array}{c} {\|{\hat{𝐩}}^{\left(\infty \right)}-{\hat{𝐩}}^{\left(n\right)}\|}_{\text{2}}\leq \sqrt{\text{3}}\;\}i\;\}d^{\left(0\right)}{\text{2}}^{1-n}, \end{array}$$ (28)

which provides a geometrically decreasing upper bound. In the implemented algorithm, the stopping criterion may terminate the refinement early; otherwise, $K_{max}$ guarantees termination after a finite number of iterations.

This proposition establishes convergence of the fitness values and the iterative position sequence. It does not guarantee convergence to the global maximizer unless the compressed initial region contains the relevant attraction region of the global solution.

#### 2.3.3 Selection of grid coverage factor.

In the adaptive iterative refinement strategy, the grid coverage factor (i), as a key parameter, has a significant impact on the localization accuracy and convergence performance of the algorithm. To systematically evaluate its influence, the localization performance of the FDCMR-AIR-DPD method was evaluated with i=1,2,3,4. The SNR was varied from −15 dB to 25 dB in increments of 5 dB, and all other simulation parameters were kept the same as those described in Section 3.1. For each combination of the coverage factor and SNR level, 500 independent Monte Carlo trials were performed. The 95% confidence intervals of the RMSE estimates were calculated using the percentile-bootstrap method with 10,000 resamples. The results are shown in Fig 3.

**Fig 3:**
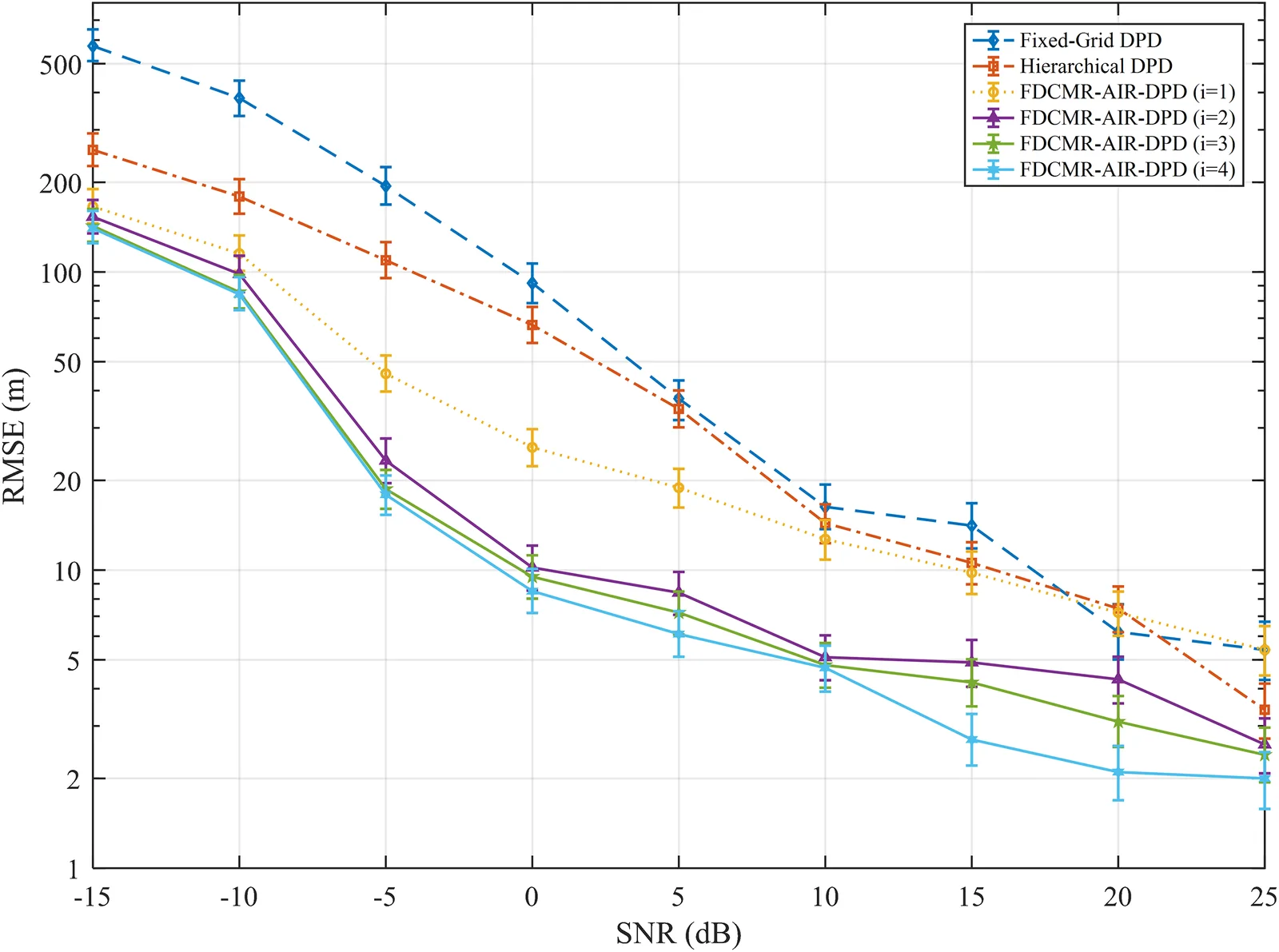
Selection of the coverage factor (i). RMSE comparison under different coverage factors and SNR levels. Error bars denote the 95% bootstrap confidence intervals obtained from 500 independent Monte Carlo trials and 10,000 bootstrap resamples.

The coverage factor i determines the size of the local voxel search region in each adaptive iteration. For a given i, the number of candidate voxels evaluated at each iteration is $(2i+1{)}^{\text{3}}$. Therefore, increasing i can enlarge the local search region and improve the probability of covering the optimal refinement direction, but it also increases the DPD evaluation cost cubically.

As shown in Fig 3, the RMSE curves and their 95% bootstrap confidence intervals exhibit consistent overall performance trends under different coverage factors. When i increases from 1 to 2, the localization RMSE is substantially reduced, especially under low-SNR conditions. This result suggests that i=1 provides an insufficient local search range and may fail to cover the optimal refinement direction when the coarse estimate is affected by noise. However, when i is further increased from 2 to 3 or 4, the additional reduction in RMSE becomes limited. In contrast, the number of candidate voxels increases from 125 for i=2 to 343 for i=3 and 729 for i=4. This means that the DPD evaluation cost becomes approximately 2.744 and 5.832 times that for i=2, respectively.

Therefore, under the considered simulation conditions, i=2 is selected as a favorable accuracy-complexity tradeoff rather than the minimum-RMSE setting. This choice provides sufficient robustness for adaptive local refinement while avoiding the unnecessary computational burden caused by an excessively large search region

### 2.4 Difference from conventional search strategies

Although the proposed method adopts a coarse-to-fine localization framework, it differs fundamentally from conventional hierarchical grid search and multi-resolution DPD in terms of search-space construction, DPD evaluation region, and resolution update strategy.


$$\begin{array}{c} \Omega_{s}=B\left({\hat{𝐩}}_{s-1},r_{s}\right), \end{array}$$ (29)

$$\begin{array}{c} {\hat{𝐩}}_{s}={argmax}_{𝐩\in \Omega_{s}}J\left(𝐩\right), \end{array}$$ (30)

where $\Omega_{s}$ denotes the search region at the s -th level,$B(\hat{p}s-1,r_{s})$ denotes a local region centered at the previous estimate with radius $r_{s}$, and J(𝐩) is the objective function. Therefore, the refined search region directly depends on the previous coarse-grid estimate. An inaccurate coarse-grid estimate may mislead the subsequent refinement stage, whereas a dense coarse grid increases the computational burden.

In multi-resolution DPD, the DPD objective function is evaluated at multiple grid resolutions:


$$\begin{array}{c} r_{sr}={argmax}_{𝐩\in \Omega_{r}}J_{\text{DPD}}(𝐩;\Delta_{r}), \end{array}$$ (31)

where $\Delta_{r}$ is the (r)-th grid resolution and $\Omega_{r}$ is the corresponding search region. Although this strategy reduces the number of grid points compared with exhaustive fine-grid DPD, the DPD objective may still be evaluated over global or semi-global regions at several resolutions. Thus, its complexity reduction mainly results from changing grid spacing rather than excluding unlikely spatial regions before DPD evaluation.

In contrast, the proposed method reduces the search space before DPD-based refinement. FDCMR-based DOA estimation is first performed at multiple receiving stations. Each DOA estimate defines a directional cone $C_{l}$ in the three-dimensional space. The compressed candidate region is obtained as:


$$\begin{array}{c} \Omega_{c}=\Omega_{o}\cap \left({⋂}_{l=1}^{L}C_{l}\right), \end{array}$$ (32)

where $\Omega_{o}$ is the original global search region, $\Omega_{c}$ is the cone-intersection candidate region, and L is the number of receiving stations. After this DOA-constrained compression, the DPD objective function is evaluated only inside $\Omega_{c}$.

The proposed adaptive voxel iteration further refines the estimate within the compressed region. At the k -th iteration, the voxel resolution follows an exponential decay law:


$$\begin{array}{c} d^{\left(k-1\right)}=\frac{d^{\left(0\right)}}{{\text{2}}^{k-1}}. \end{array}$$ (33)

The local search region is centered at the current estimate:


$$\begin{array}{c} \Omega_{k}=B\left({\hat{𝐩}}^{{\;}_{k-1}},id^{\left(k-1\right)}\right), \end{array}$$ (34)

and the local estimate is updated by:


$$\begin{array}{c} {\hat{𝐩}}_{k}={argmax}_{𝐩\in \Omega_{k}}J_{\text{DPD}}\left(𝐩\right), \end{array}$$ (35)

where $\Omega_{k}$ denotes the local voxel grid centered at the current estimate. With a coverage factor i, each iteration evaluates only $(2i+1{)}^{\text{3}}$ candidate points. Therefore, the number of DPD evaluations in the refinement stage is approximately:


$$\begin{array}{c} N_{\text{DPD}}^{\text{proposed}}=K(2i+1{)}^{\text{3}}, \end{array}$$ (36)

where K is the number of voxel iterations.

Thus, the essential distinction is that hierarchical grid search and multi-resolution DPD reduce complexity mainly by adjusting grid resolution during the search process, whereas the proposed method reduces the DPD search space before DPD evaluation through FDCMR-DOA cone intersection. This DOA-constrained search-space compression, combined with fixed-size local voxel refinement, constitutes the main source of computational gain.

### 2.5 Complexity analysis

This section analyzes the computational complexity of the proposed three-stage localization method and compares it with conventional global DPD, hierarchical grid search, and multi-resolution DPD to identify the source of its computational savings.

It is assumed that the total number of voxel points in the original 3D positioning area is Q, the number of receiving stations is L, and the total number of array elements in the double-layer UCA is M. The number of sampling points of the received signal is $N_{s}$, and the number of selected frequency-domain samples is $N_{f}$. Then the overall computational burden $C_{T}$ of the proposed method is composed of three parts: FDCMR-DOA estimation $C_{D}$, cone-intersection-based coarse localization $C_{O}$, and adaptive voxel DPD refinement $C_{I}$


$$\begin{array}{c} C_{T}=C_{D}\;+C_{O}+C_{I}. \end{array}$$ (37)

For each receiver, coarse DOA estimation is performed using the FDCMR method. The main computational steps are as follows. An $N_{s}$ -point FFT is applied to the signals received by M sensors, requiring $\text{O}\left(MN_{s}\;log\;N_{s}\right)$ operations. The frequency-domain covariance matrix ${𝐑}_{F}\in {\mathbb{C}}^{M\times M}$ is then constructed from $N_{f}$ selected frequency-domain samples, requiring $O\left(M^{\text{2}}N_{f}\right)$ operations. Eigenvalue decomposition of ${𝐑}_{F}$ is performed to obtain the frequency-domain noise subspace, with a complexity of $O\left(M^{\text{3}}\right)$. During the FDCMR-DOA spectral search, each angular grid point requires a quadratic-form evaluation with a complexity of $O\left(M^{\text{2}}\right)$. Therefore, searching over all $G_{az}\;G_{el}$ angular grid points requires $O\left(G_{az}\;G_{el}{\text{M}}^{\text{2}}\right)$ operations. The total computational complexity for one receiver is thus expressed as follows:


$$\begin{array}{c} O\left(MN_{s}\;log\;N_{s}\;+\;M^{\text{2}}N_{f}\;+\;M^{\text{3}}\;+\;G_{az}\;G_{el}\;M^{\text{2}}\right). \end{array}$$ (38)

For L receiver stations, the total complexity is as follows:


$$\begin{array}{c} C_{D}=L\cdot O(MN_{s}logN_{s}+M^{\text{2}}N_{f}+M^{\text{3}}+G_{a}zG_{e}lM^{\text{2}}). \end{array}$$ (39)

For cone-intersection-based coarse localization, the search region is discretized into $Q_{c}$ voxels with a spatial resolution of 50 m. For each candidate voxel and receiving station, the theoretical DOA is calculated and compared with the estimated DOA. The coarse position is then determined through confidence-weighted probability fusion. The corresponding computational complexity is expressed as


$$\begin{array}{c} C_{O}=O\left(Q_{c}LC_{s}\right), \end{array}$$ (40)

where $C_{s}$ is a constant representing the consistency-evaluation cost between one candidate voxel and one directional cone.

The iterative refinement process employs an adaptive voxel refinement strategy and performs at most $K_{max}$ iterations. At the k -th iteration, a three-dimensional uniform search grid is constructed with a search range of $\pm i\cdot d^{\left(k-1\right)}$ centered at the current position estimate ${\hat{𝐩}}^{\left(k-1\right)}$, where i is the coverage factor and $d^{\left(k-1\right)}=\frac{d^{\left(0\right)}}{{\text{2}}^{k-1}}$ is the grid resolution used at the k -th iteration. Here, $d^{\left(0\right)}$ denotes the initial grid resolution. The number of candidate positions evaluated at each iteration is fixed at $(2i+1{)}^{\text{3}}$, which makes the computational cost of each refinement iteration controllable.

At the k -th iteration, the DPD fitness function is evaluated for all $(2i+1{)}^{\text{3}}$ candidate positions. Since each fitness evaluation uses the selected frequency-domain samples, the computational complexity of a single-point DPD evaluation is $\text{O}\left(LMN_{f}\right)$. Therefore, the complexity of the k -th refinement iteration is


$$\begin{array}{c} C_{i}^{\left(k\right)}=\text{O}((2i+1{)}^{\text{3}}\cdot L\cdot M\cdot N_{f}). \end{array}$$ (41)

Because the algorithm performs at most $K_{\text{max}}$ iterations, the worst-case computational complexity of the adaptive refinement stage is


$$\begin{array}{c} C_{I}=\sum_{k=1}^{K_{max}}C_{i}^{\left(k\right)}=O(K_{\text{max}}\cdot (2i+1{)}^{\text{3}}\cdot L\cdot M\cdot N_{f}). \end{array}$$ (42)

The convergence criterion is based on the position change between adjacent iterations: the algorithm terminates early when ${\|{\hat{𝐩}}^{\left(k\right)}-{\hat{𝐩}}^{\left(k-1\right)}\|}_{\text{2}}<\varepsilon$, and the actual number of iterations is usually much less than $K_{max}$.

For comparison, conventional global DPD evaluates the DPD objective function at all Q candidate voxels, resulting in the following computational complexity:


$$\begin{array}{c} C_{g}=Q\cdot O(L\cdot M\cdot N_{f}). \end{array}$$ (43)

For hierarchical grid search, if the search process contains S grid levels and the number of candidate voxels at the s -th level is $Q_{S}$, its DPD-related complexity can be approximated as:


$$\begin{array}{c} C_{h}=O\left(\left(Q_{\text{1}}+Q_{\text{2}}+\cdots +Q_{S}\right)LMN_{f}\right). \end{array}$$ (44)

For multi-resolution DPD, if the DPD objective is evaluated at R different resolutions and the number of candidate voxels at the r -th resolution is $Q_{r}$, its complexity can be written as:


$$\begin{array}{c} C_{m}=O\left(\left(Q_{\text{1}}+Q_{\text{2}}+\cdots +Q_{R}\right)LMN_{f}\right). \end{array}$$ (45)

Although hierarchical grid search and multi-resolution DPD reduce the number of evaluated grid points compared with exhaustive fine-grid DPD, they still require repeated DPD evaluations over global or semi-global regions. In contrast, the proposed method first compresses the original search region through FDCMR-DOA estimation and cone intersection, and then performs DPD only in the local adaptive voxel region.

Thus, the total complexity can be expressed as:


$$\begin{array}{c} C_{T}=L\cdot O(MN_{s}logN_{s}+M^{\text{2}}N_{f}+M^{\text{3}}+G_{az}G_{el}M^{\text{2}})+O(Q_{c}LC_{s})+O(K(2i+1{)}^{\text{3}}LMN_{f}). \end{array}$$ (46)

Compared with these methods, the proposed method reduces the DPD search space before DPD evaluation through FDCMR-DOA estimation and cone intersection. Therefore, DPD is not performed over the entire global region, but only in the local voxel region generated during adaptive refinement. The key difference lies in the number of DPD evaluations. In global DPD, the number of DPD evaluations is Q. In the proposed adaptive voxel refinement stage, this number is reduced to:


$$\begin{array}{c} K(2i+1{)}^{\text{3}}. \end{array}$$ (47)

Thus, the reduction ratio of DPD evaluations can be approximated as:


$$\begin{array}{c} \eta \approx \frac{Q}{K(2i+1{)}^{\text{3}}}. \end{array}$$ (48)

Under the simulation setting used in this study, the global search region is 2000m×2000m×2000m, and the global DPD resolution is 5m. Thus, the number of grid points along each direction is 2000/5+1=401, and the total number of global DPD candidate voxels is $Q=401^{\text{3}}=64481201$. For the proposed adaptive voxel refinement, the maximum number of iterations is set to K=5 in the simulation experiments. According to the coverage-factor selection analysis in Section 2.3.3, the coverage factor is set to i=2, which provides a favorable balance between localization accuracy and computational cost. Therefore, the number of DPD objective evaluations in the adaptive voxel refinement stage is:


$$\begin{array}{c} K(2i+1{)}^{\text{3}}=5(2\times 2+1{)}^{\text{3}}=625. \end{array}$$ (49)

Consequently, the reduction ratio is


$$\begin{array}{c} \eta \approx 64,481,201/625\approx 1.03\times 10^{\text{5}}. \end{array}$$ (50)

This result shows that, under the same search region and spatial resolution, the proposed method reduces the number of DPD objective evaluations in the spatial search stage by approximately five orders of magnitude compared with conventional global DPD. This substantial reduction in the dominant DPD search burden is achieved because the FDCMR-DOA cone-intersection step compresses the original global search region before DPD-based adaptive voxel refinement

## 3 Simulation experiments

### 3.1 Overall positioning performance analysis

As shown in Fig 4, the simulation scenario consists of a cubic search region measuring 2000 m × 2000 m × 2000 m. Four receiving stations are located at the four corners of the ground plane. Each station is equipped with a double-layer UCA comprising 12 upper-layer elements and 8 lower-layer elements. A single stationary interference source operating at the BeiDou B1I carrier frequency of 1561.098 MHz is located at (1000 m, 1000 m, 1000 m). The SNR is set to 15 dB, and the grid coverage factor is set to i=2. The estimated source position is (1000.8 m, 999.4 m, 1001.5 m), demonstrating that the proposed method can accurately locate the interference source under the considered conditions.

**Fig 4:**
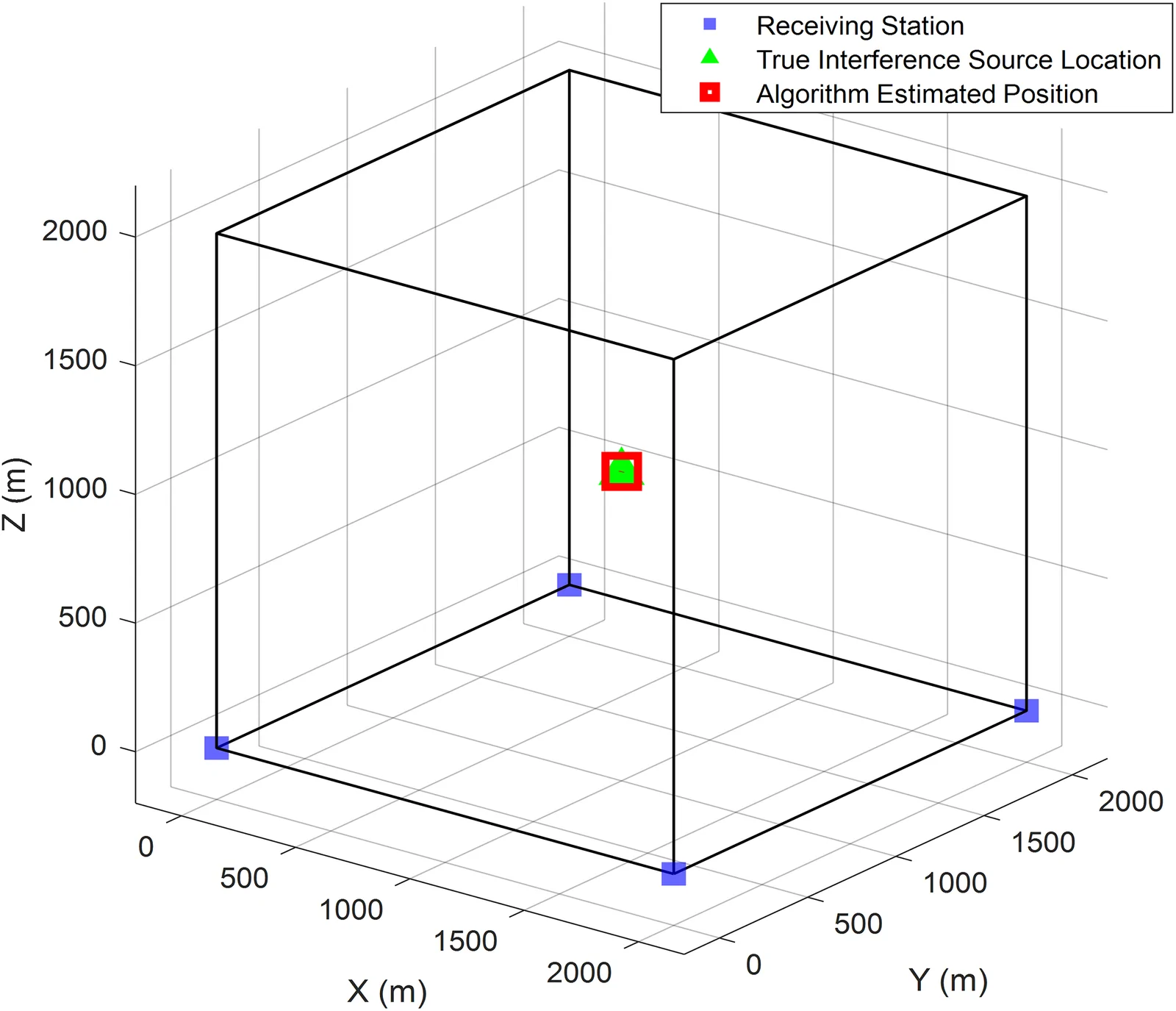
Static jamming source location result. The figure presents the final three-dimensional positioning results of the proposed method for a stationary interference source scenario.

To evaluate the spatial variation in localization accuracy, additional simulations are conducted at five representative altitude levels: 250 m, 750 m, 1250 m, 1750 m, and 2000 m. The SNR is fixed at 20 dB, while the other simulation parameters remain unchanged. At each altitude, 500 interference-source positions are uniformly generated in the horizontal plane, and 300 independent Monte Carlo trials are performed for each test position. The resulting localization-error distributions and statistical results are presented in Fig 5 and Table 1, respectively.

**Fig 5:**
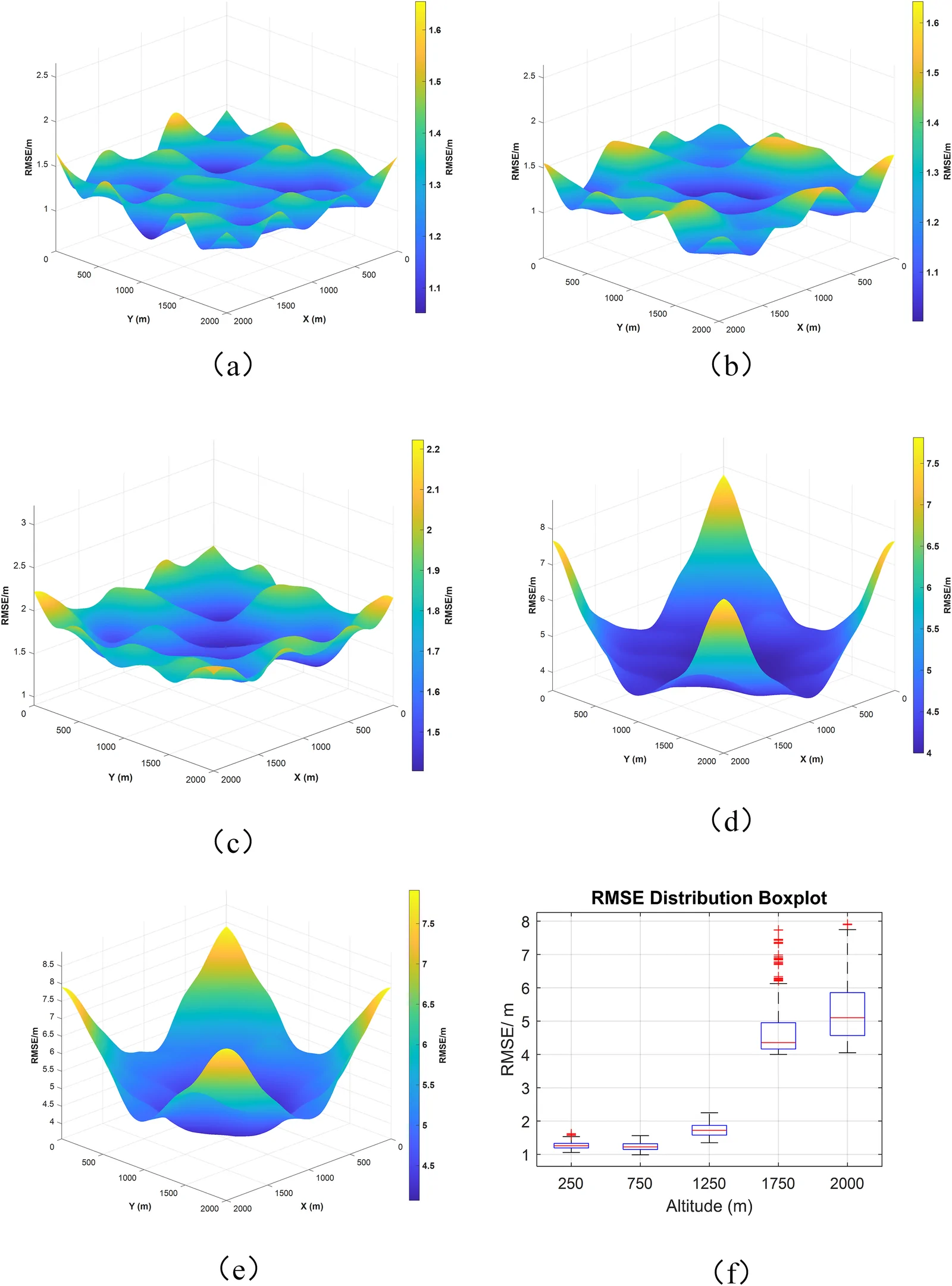
Localization-error distributions at different altitudes. Surface plots at altitudes of (a) 250 m, (b) 750 m, (c) 1250 m, (d) 1750 m, and (e) 2000 m; (f) box plots of the localization errors at the five altitudes.

**Table 1:** Statistical results of positioning accuracy at different altitude levels.

Altitude/m	RMSE/m	Error Range/m
250	1.28	1.06–1.54
750	1.24	0.99–1.63
1250	1.72	1.42–2.30
1750	4.71	4.00–7.80
2000	5.30	4.05–7.91

The quantitative results are summarized in Table 1 and show a clear dependence of localization accuracy on source altitude. At altitudes of 250 m and 750 m, the RMSE values are 1.28 m and 1.24 m, respectively, indicating relatively uniform and accurate localization at low altitudes. At 1250 m, the RMSE increases to 1.72 m, and the localization-error distribution exhibits stronger spatial dependence. The central region maintains relatively high localization accuracy, whereas larger errors occur near the boundaries because of the less favorable source-station geometry. At altitudes of 1750 m and 2000 m, the RMSE values increase to 4.71 m and 5.30 m, respectively, indicating that localization performance degrades as the source altitude increases. Overall, the proposed method provides better localization performance in the low- and medium-altitude regions. Approximately 75% of the test points have localization errors below 3 m.

### 3.2 Comparison of algorithm performance

To verify the effectiveness of the proposed FDCMR-AIR-DPD algorithm, the interference source is randomly distributed in the positioning region under the experimental conditions described in Section 3.1. The SNR is varied from −15 dB to 25 dB in steps of 5 dB, and 500 Monte Carlo trials are performed at each SNR level. For each algorithm and SNR level, the RMSE is calculated from the localization errors obtained in the 500 independent Monte Carlo trials. To quantify the statistical uncertainty of the RMSE estimates, a nonparametric percentile-bootstrap method is employed. Specifically, 10,000 bootstrap samples, each containing 500 localization errors drawn with replacement from the original Monte Carlo results, are generated. The RMSE is recalculated for each bootstrap sample, and the 2.5th and 97.5th percentiles of the bootstrap RMSE distribution are used as the lower and upper bounds of the 95% confidence interval, respectively. The proposed method is compared with the conventional two-step method, fixed-grid DPD, and maximum-likelihood DPD (ML-DPD). The RMSE curves with 95% bootstrap confidence intervals are shown in Fig 6.

**Fig 6:**
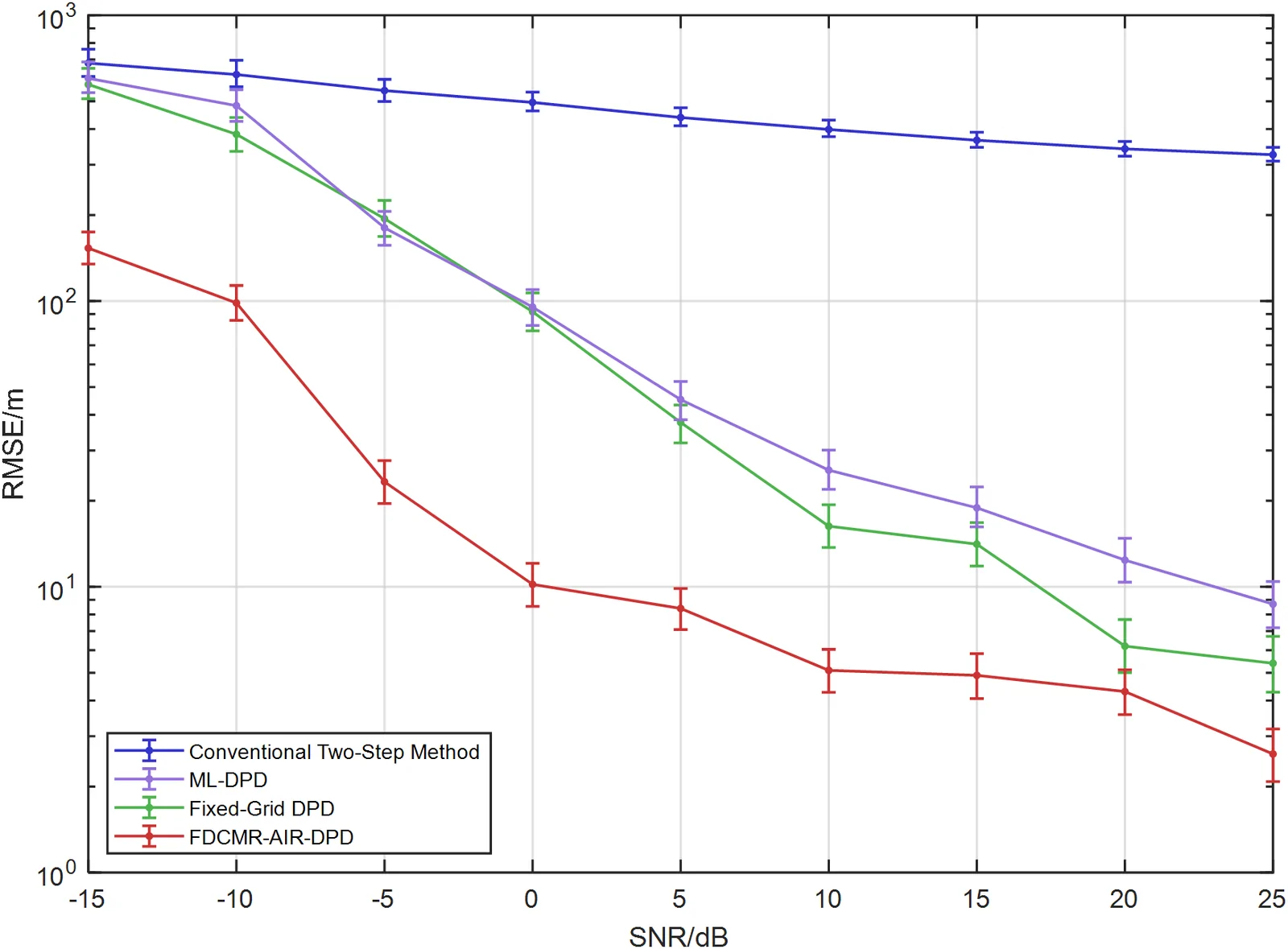
Localization RMSEs of different methods with 95% bootstrap confidence intervals. The proposed FDCMR-AIR-DPD method is compared with the conventional two-step method, ML-DPD, and fixed-grid DPD under different SNR levels. The error bars denote the percentile-bootstrap 95% confidence intervals calculated using 10,000 bootstrap resamples of the localization errors obtained from 500 Monte Carlo trials.

As shown in Fig 6, the RMSE-versus-SNR curves with 95% bootstrap confidence intervals show clear performance differences among the four algorithms. The error bars quantify the uncertainty in the RMSE estimates caused by finite Monte Carlo sampling. Under low-SNR conditions from −15 dB to −5 dB, the proposed FDCMR-AIR-DPD method exhibits strong robustness to noise. Its RMSE decreases from 153.2 m at −15 dB to 23.3 m at −5 dB and remains lower than those of the conventional two-step method, ML-DPD, and fixed-grid DPD.

As the SNR increases to the moderate range from 0 dB to 10 dB, the localization accuracy of all four methods improves. However, the proposed method continues to achieve a lower RMSE, decreasing from 10.2 m at 0 dB to 5.1 m at 10 dB. In the high-SNR range from 15 dB to 25 dB, the proposed method maintains the lowest localization error among the compared methods, with its RMSE remaining below 5 m and reaching 2.6 m at 25 dB. These results demonstrate that the proposed method provides consistently improved localization accuracy over the considered SNR range.

### 3.3 Comparison with the Cramer-Rao bound

To evaluate the statistical efficiency of the proposed low-complexity localization method, its RMSE is compared with the deterministic Cramer-Rao bound (CRB). The deterministic CRB provides a theoretical lower bound on the covariance of any unbiased estimator when the unknown source position and channel parameters are treated as fixed but unknown quantities. It therefore provides a benchmark for evaluating the gap between the proposed method and the theoretical lower bound under the specified observation model and noise conditions.

Let ${𝐩}_{s}=[x_{s},y_{s},z_{s}{]}^{T}$ denote the unknown true three-dimensional position of the interference source, and let ${𝐩}_{l}=[x_{l},y_{l},z_{l}{]}^{T}$ denote the position of the l -th receiving station. For the l -th receiving station and the k -th selected frequency bin, the frequency-domain received signal is expressed as


$$\begin{array}{c} {𝐱}_{l,k}=\mu_{l,k}+{𝐧}_{l,k}=\alpha_{l}S\left[k\right]{𝐚}_{l}\left(\theta_{l},\phi_{l}\right)e^{-j2\pi f_{k}\tau_{l}}+{𝐧}_{l,k}, \end{array}$$ (51)

where $\alpha_{l}$ is the unknown complex path gain of the l -th receiving station, S[k] is the deterministic spectral coefficient at the k -th selected frequency bin, ${𝐚}_{l}\left(\theta_{l},\phi_{l}\right)\in {\mathbb{C}}^{M\times 1}$ is the double-layer UCA steering vector, $f_{k}$ is the corresponding frequency, and $\tau_{l}$ is the propagation delay. The noise vector satisfies ${𝐧}_{l,k}\sim 𝒞𝒩\left(0,\sigma^{\text{2}}{𝐈}_{M}\right)$, where 𝒞𝒩(·,·) denotes the proper complex Gaussian distribution, $\sigma^{\text{2}}$ is the noise variance of each antenna element, and ${𝐈}_{M}$ is the M×M identity matrix.

Consistent with the matched-processing model, S[k] is treated as known when evaluating the deterministic CRB, whereas the complex path gains are treated as deterministic nuisance parameters.

After stacking the observations from all L receiving stations and all $N_{f}$ selected frequency bins, the complete observation model is written as 𝐱=μ(η)+𝐧, where μ(η) is the stacked noise-free observation vector. The complete deterministic parameter vector is defined as


$$\begin{array}{c} \eta =[x_{s},y_{s},z_{s},\alpha_{1,R},\alpha_{1,I},\ldots ,\alpha_{L,R},\alpha_{L,I}{]}^{T}, \end{array}$$ (52)

where $\alpha_{l,R}=Re\left\{\alpha_{l}\right\}$,$\alpha_{l,I}=Im\left\{\alpha_{l}\right\}$. Because the noise covariance is independent of η, the Fisher information matrix (FIM) under the complex AWGN model is given by


[J(η)]m,n=2σ2Re∂μ∂ηmH∂μ∂ηn, (53)

where $\eta_{m}$ and $\eta_{n}$ are the m -th and n -th elements of η, respectively, and $(\cdot {)}^{H}$ denotes the Hermitian transpose.

For $q\in \left\{x_{s},y_{s},z_{s}\right\}$, the derivative of the noise-free observation with respect to the source-position parameter q is obtained using the chain rule:


$$\begin{array}{c} \frac{\partial \mu_{l,k}}{\partial q}=\alpha_{l}S\left[k\right]e^{-j2\pi f_{k}\tau_{l}}\left[\frac{\partial {𝐚}_{l}}{\partial \theta_{l}}\frac{\partial \theta_{l}}{\partial q}+\frac{\partial {𝐚}_{l}}{\partial \phi_{l}}\frac{\partial \phi_{l}}{\partial q}-j2\pi f_{k}{𝐚}_{l}\frac{\partial \tau_{l}}{\partial q}\right]. \end{array}$$ (54)

The relative geometric quantities between the source and the l -th receiving station are defined as


$$\begin{array}{c} \begin{array}{c} \Delta x_{l}=x_{s}-x_{l},\;\;\}\Delta y_{l}=y_{s}-y_{l},\;\;\}\Delta z_{l}=z_{s}-z_{l},\\ r_{l}=\sqrt{\Delta x_{l}^{\text{2}}+\Delta y_{l}^{\text{2}}+\Delta z_{l}^{\text{2}}},\;\;\}\rho_{l}=\sqrt{\Delta x_{l}^{\text{2}}+\Delta y_{l}^{\text{2}}}. \end{array} \end{array}$$ (55)

Accordingly, the propagation delay, azimuth angle, and pitch angle are expressed as


$$\begin{array}{c} \tau_{l}=\frac{r_{l}}{c},\;\;\}\theta_{l}=atan2\left(\Delta y_{l},\Delta x_{l}\right),\;\;\}\phi_{l}=atan2\left(\Delta z_{l},\rho_{l}\right), \end{array}$$ (56)

where c is the speed of light. The required derivatives of the propagation delay are


$$\begin{array}{c} \frac{\partial \tau_{l}}{\partial x_{s}}=\frac{\Delta x_{l}}{cr_{l}},\;\;\}\frac{\partial \tau_{l}}{\partial y_{s}}=\frac{\Delta y_{l}}{cr_{l}},\;\;\}\frac{\partial \tau_{l}}{\partial z_{s}}=\frac{\Delta z_{l}}{cr_{l}}. \end{array}$$ (57)

The derivatives of the azimuth angle are


$$\begin{array}{c} \frac{\partial \theta_{l}}{\partial x_{s}}=-\frac{\Delta y_{l}}{\rho_{l}^{\text{2}}},\;\;\}\frac{\partial \theta_{l}}{\partial y_{s}}=\frac{\Delta x_{l}}{\rho_{l}^{\text{2}}},\;\;\}\frac{\partial \theta_{l}}{\partial z_{s}}=0. \end{array}$$ (58)

The derivatives of the pitch angle are


$$\begin{array}{c} \frac{\partial \phi_{l}}{\partial x_{s}}=-\frac{\Delta x_{l}\Delta z_{l}}{r_{l}^{\text{2}}\rho_{l}},\;\;\}\frac{\partial \phi_{l}}{\partial y_{s}}=-\frac{\Delta y_{l}\Delta z_{l}}{r_{l}^{\text{2}}\rho_{l}},\;\;\}\frac{\partial \phi_{l}}{\partial z_{s}}=\frac{\rho_{l}}{r_{l}^{\text{2}}}. \end{array}$$ (59)

For the complex path-gain nuisance parameters, the corresponding derivatives are


$$\begin{array}{c} \frac{\partial \mu_{l,k}}{\partial \alpha_{r,R}}=\delta_{lr}S\left[k\right]{𝐚}_{l}\left(\theta_{l},\phi_{l}\right)e^{-j2\pi f_{k}\tau_{l}}, \end{array}$$ (60)

$$\begin{array}{c} \frac{\partial \mu_{l,k}}{\partial \alpha_{r,I}}=j\delta_{lr}S\left[k\right]{𝐚}_{l}\left(\theta_{l},\phi_{l}\right)e^{-j2\pi f_{k}\tau_{l}}, \end{array}$$ (61)

where $\delta_{lr}$ is the Kronecker delta. The derivatives of the double-layer UCA steering vector with respect to $\theta_{l}$ and $\phi_{l}$ are obtained analytically from the steering-vector model introduced in Section 2.1.

The complete FIM is then partitioned according to the source-position parameters and the nuisance path-gain parameters:


$$\begin{array}{c} 𝐉\left(\eta \right)=\left[\begin{array}{cc} {𝐉}_{ss} & {𝐉}_{s\alpha }\\ {𝐉}_{\alpha s} & {𝐉}_{\alpha \alpha } \end{array}\right], \end{array}$$ (62)

where the subscripts s and α denote the source-position parameters and the nuisance path-gain parameters, respectively.

After eliminating the nuisance parameters through the Schur complement, the equivalent Fisher information matrix for the source position is obtained as


$$\begin{array}{c} {𝐉}_{e}\left({𝐩}_{s}\right)={𝐉}_{ss}-{𝐉}_{s\alpha }{𝐉}_{\alpha \alpha }^{-1}{𝐉}_{\alpha s}. \end{array}$$ (63)

The deterministic CRB matrix of the source position is therefore


$$\begin{array}{c} {𝐂}_{\text{CRB}}\left({𝐩}_{s}\right)={𝐉}_{e}^{-1}\left({𝐩}_{s}\right). \end{array}$$ (64)

The corresponding three-dimensional RMSE lower bound for a fixed source position is


$$\begin{array}{c} {\text{RMSE}}_{\text{CRB}}\left({𝐩}_{s}\right)=\sqrt{tr\left[{𝐂}_{\text{CRB}}\left({𝐩}_{s}\right)\right]}. \end{array}$$ (65)

Here, tr(·) denotes the matrix trace.Because the source position varies among the $N_{\text{MC}}$ Monte Carlo trials, the deterministic CRB corresponding to the overall RMSE is calculated as


$$\begin{array}{c} {\overset{―}{\text{RMSE}}}_{\text{CRB}}=\sqrt{\frac{\text{1}}{N_{\text{MC}}}\sum_{r=1}^{N_{\text{MC}}}tr\left[{𝐂}_{\text{CRB}}\left({𝐩}_{s}^{\left(r\right)}\right)\right]}. \end{array}$$ (66)

where $N_{\text{MC}}=500$ is the number of Monte Carlo trials and ${𝐩}_{s}^{\left(r\right)}$ is the true source position in the r -th trial.

In this study, the deterministic CRB is numerically evaluated using the same base-station geometry, double-layer UCA configuration, selected frequency bins, source positions, and AWGN conditions as those used in the Monte Carlo simulations. The unknown complex path gains are incorporated as deterministic nuisance parameters and eliminated through the Schur complement. The noise variance at each SNR level is determined using the same SNR definition as that employed in the localization simulations.

As shown in Fig 7, the RMSE of the proposed FDCMR-AIR-DPD method remains above the deterministic CRB over the entire SNR range and follows the same decreasing trend as the theoretical bound. In the low-SNR region, a relatively large gap is observed because noise-induced DOA estimation errors can result in inaccurate direction-cone intersections and coarse-position estimates. In the medium-SNR region, the gap decreases as the DOA estimation and adaptive refinement become more reliable. At high SNR levels, the deterministic CRB continues to decrease, whereas the RMSE of the proposed method decreases more slowly because of voxel discretization, residual DOA estimation errors, and the finite number of adaptive iterations. These results indicate that the proposed method achieves a reasonable balance between localization accuracy and computational complexity while maintaining the expected relationship with the theoretical lower bound.

**Fig 7:**
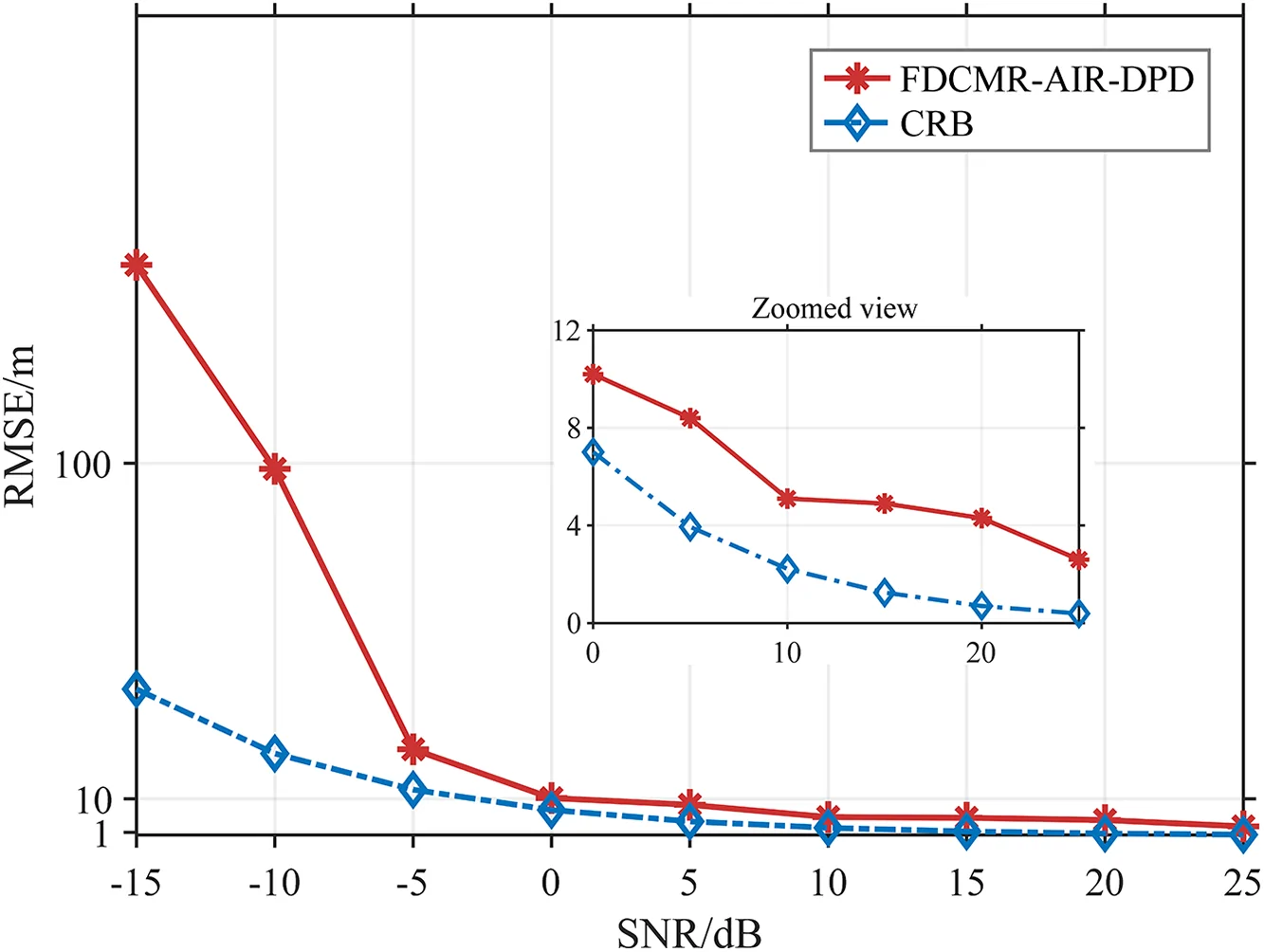
RMSE comparison with the deterministic CRB. The inset shows a zoomed view of the medium- and high-SNR regions.

### 3.4 Effectiveness of adaptive iterative refinement

To verify the effectiveness of the adaptive iterative refinement strategy, three algorithms are compared: FDCMR-AIR-DPD, FDCMR-Hierarchical-DPD, and FDCMR-Fixed-Grid DPD. The experiment is conducted under the conditions described in Section 3.1, and 500 Monte Carlo trials are performed at each SNR level. The results are shown in Fig 8.

**Fig 8:**
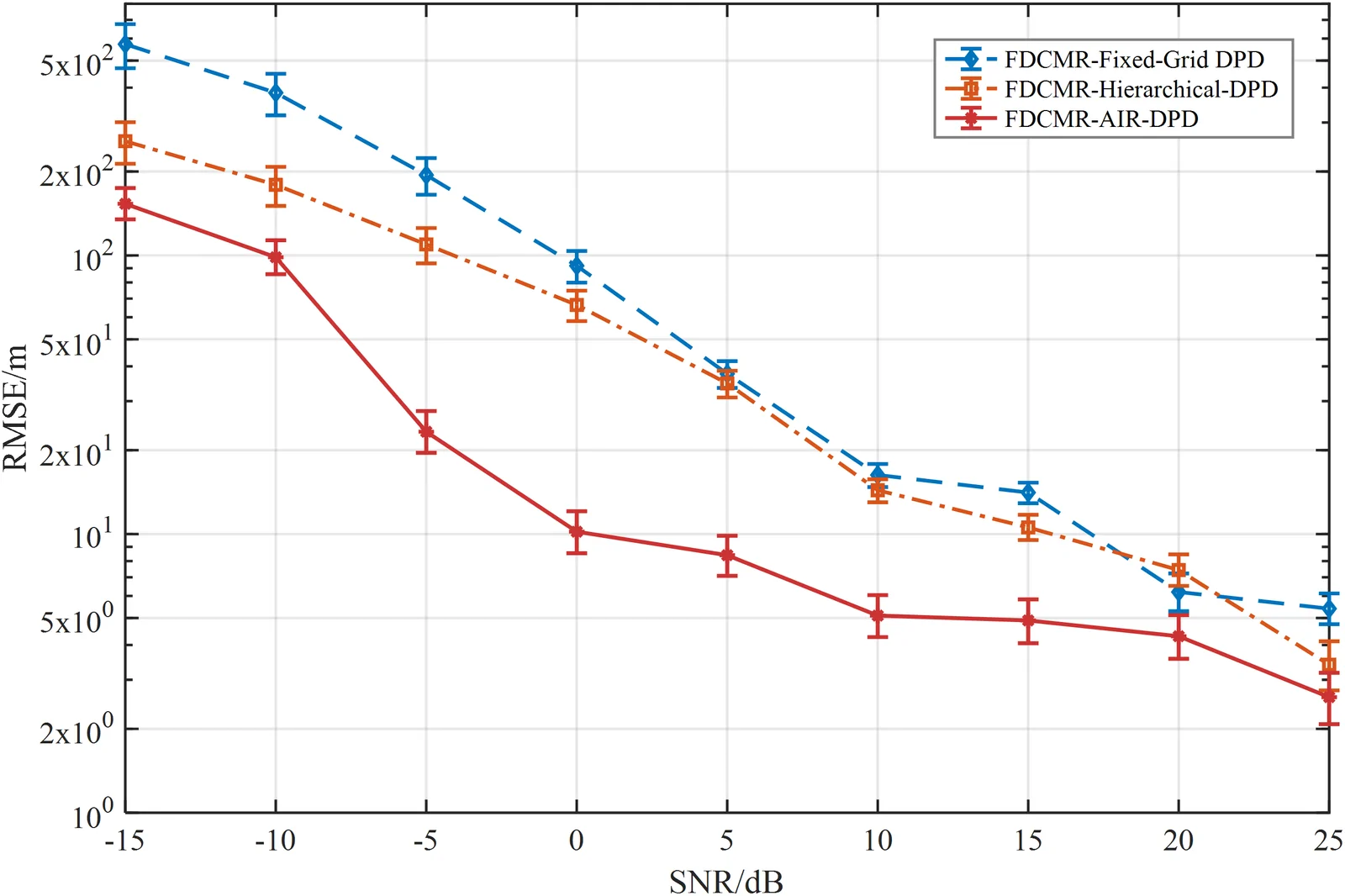
Performance comparison of different refinement strategies. RMSE comparison of the FDCMR-Fixed-Grid DPD, FDCMR-Hierarchical-DPD, and proposed FDCMR-AIR-DPD methods under different SNR levels. Error bars denote the 95% bootstrap confidence intervals obtained from 500 Monte Carlo trials and 10,000 bootstrap resamples.

As shown in Fig 8, the three algorithms show clear performance differences. In the low-SNR range from −15 dB to −5 dB, the fixed-grid DPD algorithm suffers from relatively large localization errors because of the fixed grid resolution. The hierarchical DPD algorithm reduces the RMSE through a multi-resolution search strategy. In contrast, the proposed FDCMR-AIR-DPD method achieves lower RMSE by combining FDCMR-DOA constrained coarse localization with adaptive local refinement. In the high-SNR range from 15 dB to 25 dB, all algorithms improve, while the proposed method still maintains the lowest RMSE. This result supports the effectiveness of the adaptive refinement strategy.

### 3.5 Analysis of single-station DOA error

To evaluate the robustness of the FDCMR method using a double-layer UCA at a single station, the SNR is set to −10 dB under the experimental configuration described in Section 3.1. A single double-layer UCA base station is used. The pitch angle is varied from 10° to 80°, and the azimuth angle is varied from 0° to 90°, with a step size of 2°.

As shown in Fig 9, the pitch-angle estimation results agree well with the actual values, and the data points are close to the ideal diagonal. The pitch-angle error mostly remains within 2°, with slight increases near the low- and high-elevation regions. The azimuth-angle estimation results also show good agreement with the actual values, and the azimuth error remains within 1° over the tested angular range. This confirms that the FDCMR method provides stable single-station DOA estimation under low-SNR conditions.

**Fig 9:**
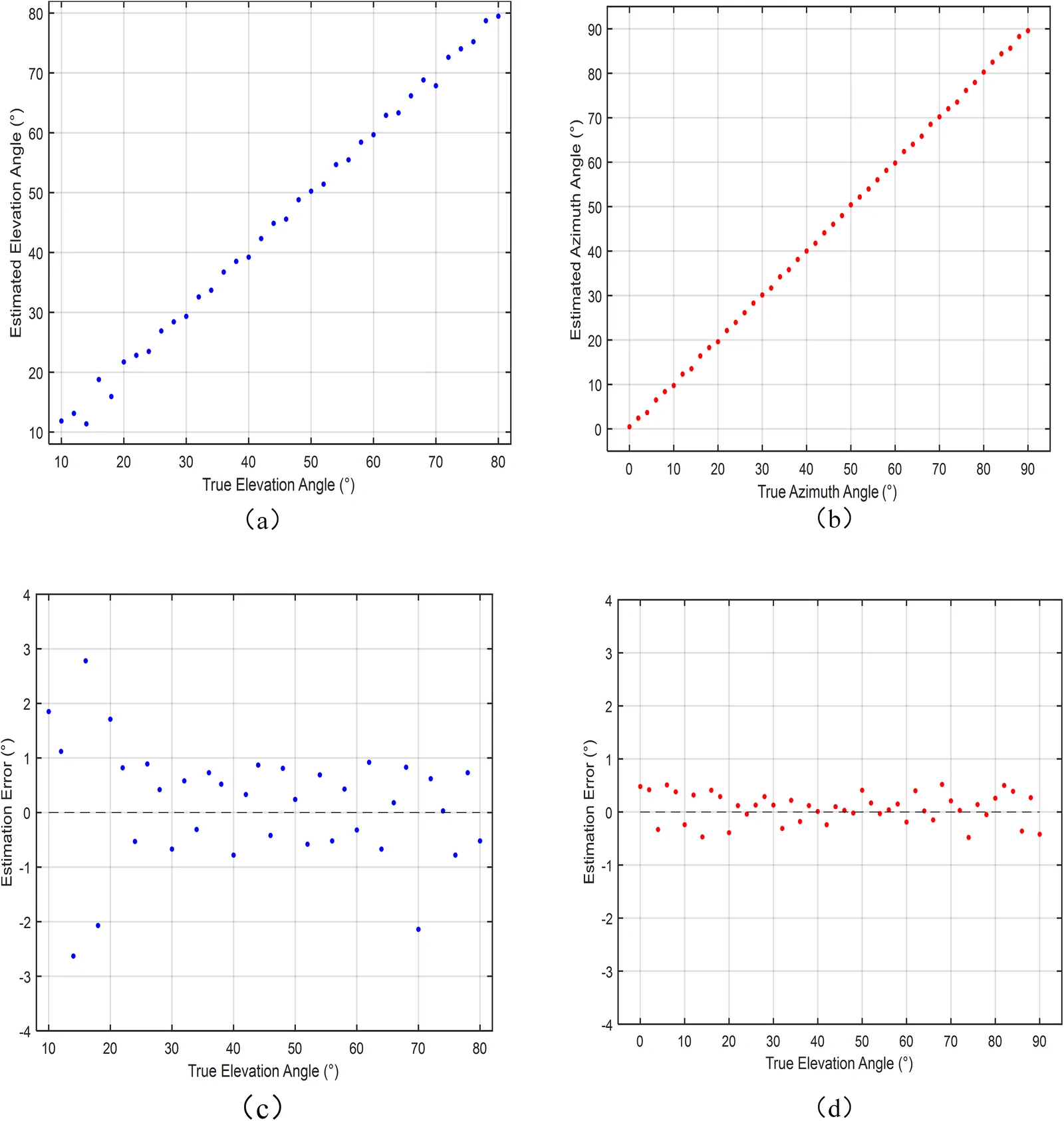
DOA estimation performance at different incidence angles. (a) Pitch-angle estimates; (b) azimuth-angle estimates; (c) pitch-angle errors; and (d) azimuth-angle errors.

### 3.6 Analysis of the four-station constraint success rate

To verify the four-station constraint success rate under different SNR conditions, the SNR is varied from −15 dB to 10 dB with a step size of 2 dB. Under the configuration described in Section 3.1, 300 independent Monte Carlo trials are performed at each SNR level. The FDCMR algorithm is compared with MUSIC, estimation of signal parameters via rotational invariance techniques (ESPRIT), and compressive-sensing-based DOA (CS-DOA) algorithms using the double-layer UCA.

As shown in Fig 10, the FDCMR-based four-station constraint method achieves a higher success rate than the other three DOA estimation algorithms under low-SNR conditions. When the SNR is below 0 dB, the DOA estimation accuracy of the traditional MUSIC and ESPRIT algorithms decreases rapidly, which leads to a significant decline in the success rate of the four-station constraint. When the SNR is lower than −11 dB, the success rates of MUSIC and ESPRIT decrease to less than 0.2. Although the CS-DOA algorithm has the advantage of relatively low computational cost, its accuracy is also seriously degraded under low-SNR conditions. In contrast, the FDCMR method maintains a higher success rate over a wider SNR range, demonstrating its robustness for multi-station geometric constraint localization.

**Fig 10:**
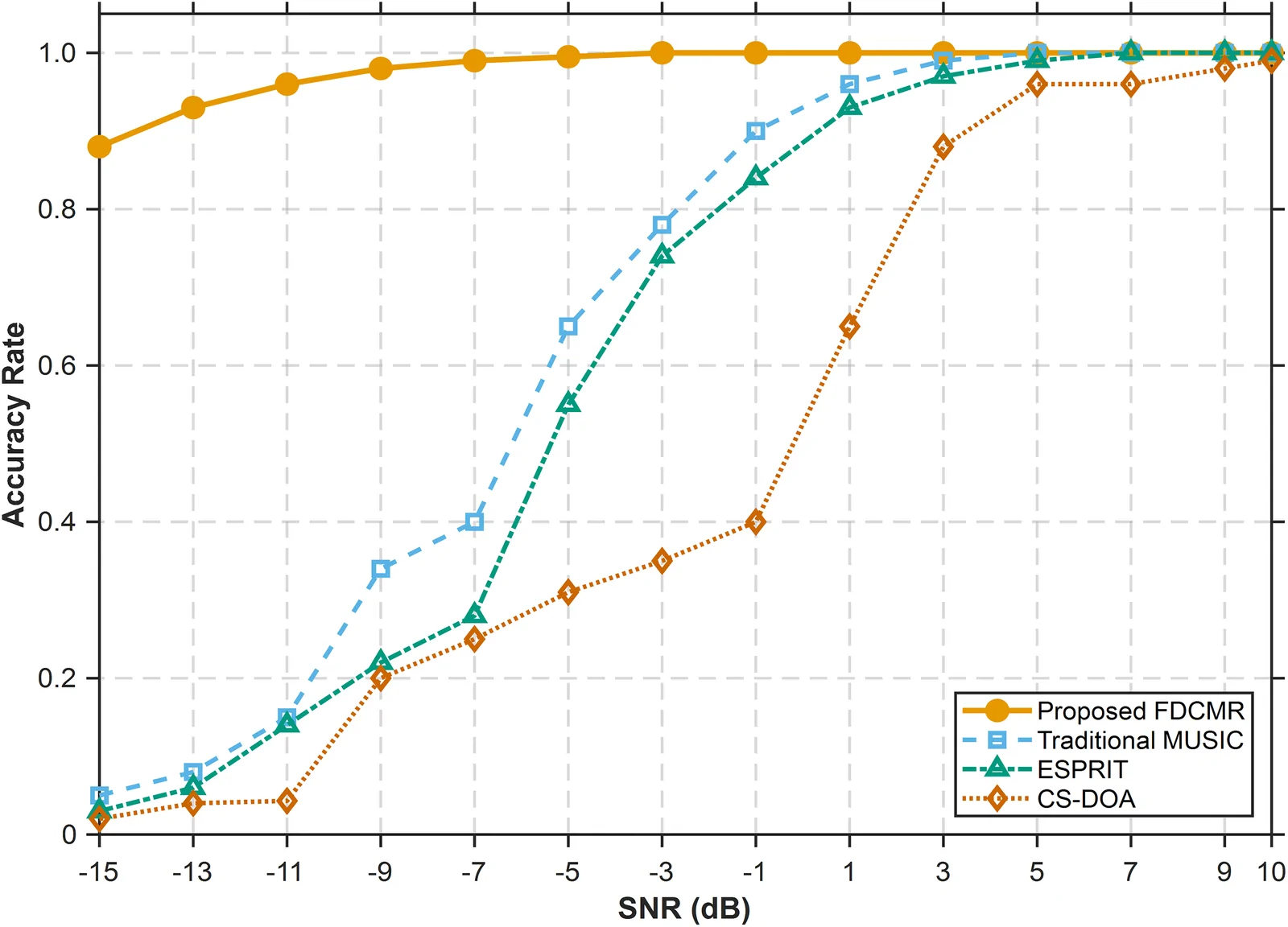
Success rate of four-station constraint. Success rate comparison of four DOA estimation algorithms under a four-station geometric constraint across varying SNR.

### 3.7 Influence of inter-layer spacing

The vertical distance between the upper and lower UCA layers has an important influence on the pitch-angle estimation and the final localization accuracy. According to the double-layer UCA steering-vector model, the vertical phase difference introduced by the inter-layer spacing h is proportional to $\frac{2\pi }{\lambda }hsin\phi$. Therefore, increasing h can enlarge the vertical aperture and improve the sensitivity of the array response to the pitch angle. However, an excessively large h may also increase the risk of phase ambiguity and make the steering-vector matching more sensitive to noise and model errors.

To evaluate this effect, the localization performance was tested under different normalized inter-layer spacings h/λ. The value of h/λ was varied from 1 to 10, and three representative SNR levels, namely −10 dB, 5 dB, and 15 dB, were considered. Other simulation parameters were kept the same as those in Section 3.1. For each combination of inter-layer spacing and SNR, 500 independent Monte Carlo trials were performed. The error bars represent the 95% percentile-bootstrap confidence intervals of the RMSE estimates obtained using 10,000 bootstrap resamples.

Fig 11 shows the variation in RMSE with h/λ under different SNR levels. When h/λ is small, the limited vertical aperture results in insufficient vertical phase discrimination and relatively large localization errors. As h/λ increases, the RMSE generally decreases because the enlarged vertical aperture improves pitch-angle resolution. Under the considered simulation conditions, the lowest mean RMSE for all three SNR levels is obtained at h/λ=6, where the RMSE values are 98.5 m, 8.4 m, and 4.9 m at (−10) dB, (5) dB, and (15) dB, respectively.

**Fig 11:**
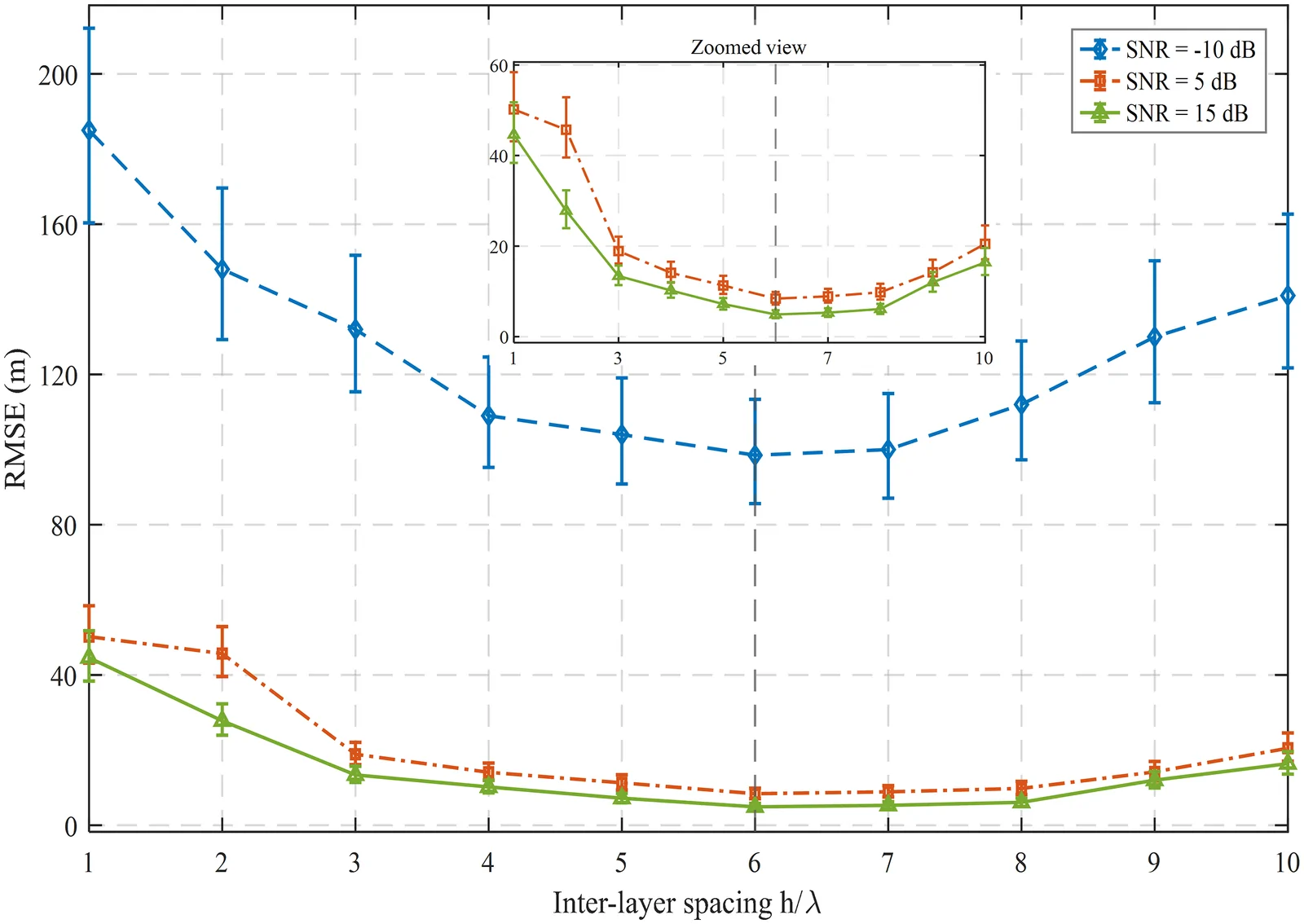
Influence of normalized inter-layer spacing on localization accuracy. Error bars denote the 95% percentile-bootstrap confidence intervals obtained from 500 Monte Carlo trials and 10,000 bootstrap resamples.

When h/λ is increased beyond 6, the mean RMSE no longer decreases and gradually increases. This degradation is mainly attributed to the increased risk of phase ambiguity and the enhanced sensitivity of steering-vector matching to noise and model mismatch. Although some confidence intervals at adjacent spacing values may overlap, h/λ=6 provides the lowest overall mean RMSE across the three tested SNR levels. Therefore, it is selected as an empirical balance between pitch-angle resolution and phase-ambiguity risk under the considered array configuration and simulation conditions.

### 3.8 Robustness to residual synchronization errors

The signal model established in Section 2.1 assumes ideal time-frequency synchronization among the receiving stations. In practical distributed localization systems, however, residual timing and carrier-frequency offsets may remain because of sampling-clock mismatch, oscillator instability, and incomplete synchronization compensation. To evaluate the robustness of the proposed method under these synchronization imperfections, additional simulations were conducted by independently introducing residual synchronization errors at different receiving stations.

For the l -th receiving station, the received signal containing residual synchronization errors is expressed as


$$\begin{array}{c} {𝐱}_{l}\left[n\right]=\alpha_{l}{𝐚}_{l}\left(\theta_{l},\phi_{l}\right)s\left(nT_{s}-\tau_{l}-\Delta \tau_{l}\right)e^{j2\pi \Delta f_{l}nT_{s}}+{𝐧}_{l}\left[n\right], \end{array}$$ (67)

where ${𝐱}_{l}\left[n\right]$ denotes the received signal vector in the presence of residual synchronization errors, $\Delta \tau_{l}$ is the residual timing offset, and $\Delta f_{l}$ is the residual carrier-frequency offset of the l -th receiving station. The other symbols follow the definitions given in Section 2.1.

The normalized timing-offset level is defined as


$$\begin{array}{c} \varepsilon_{\tau }=\frac{\Delta \tau_{max}}{T_{s}}, \end{array}$$ (68)

where $\Delta \tau_{max}$ is the maximum absolute timing offset and $T_{s}$ is the sampling interval. In each Monte Carlo trial, the residual timing offset of the l -th receiving station is independently generated according to


$$\begin{array}{c} \Delta \tau_{l}\;𝒰\left(-\varepsilon_{\tau }T_{s},\;\}\varepsilon_{\tau }T_{s}\right). \end{array}$$ (69)

where 𝒰(a,b) denotes the continuous uniform distribution over the interval [a,b].The normalized carrier-frequency-offset level is defined as


$$\begin{array}{c} \varepsilon_{f}=\frac{\Delta f_{max}}{\Delta f_{\text{bin}}}, \end{array}$$ (70)

where $\Delta f_{max}$ is the maximum absolute carrier-frequency offset and $\Delta f_{\text{bin}}$ is the FFT frequency-bin spacing, given by


$$\begin{array}{c} \Delta f_{\text{bin}}=\frac{\text{1}}{N_{s}T_{s}}. \end{array}$$ (71)

Accordingly, the residual carrier-frequency offset of the l -th receiving station is independently generated as


$$\begin{array}{c} \Delta f_{l}\;𝒰\left(-\varepsilon_{f}\Delta f_{\text{bin}},\;\}\varepsilon_{f}\Delta f_{\text{bin}}\right). \end{array}$$ (72)

The normalized timing- and carrier-frequency-offset levels were set to


$$\begin{array}{c} \varepsilon_{\tau },\varepsilon_{f}\in \left\{0,\;\}0.02,\;\}0.05,\;\}0.10,\;\}0.20,\;\}0.30\right\}. \end{array}$$ (73)

The two types of synchronization errors were evaluated separately. The carrier-frequency offset was set to zero in the timing-offset experiment, whereas the timing offset was set to zero in the carrier-frequency-offset experiment. Three representative SNR levels, namely −10, 5, and 15 dB, were considered. All other simulation parameters were kept the same as those described in Section 3.1. For each combination of SNR and synchronization-error level, 500 independent Monte Carlo trials were performed. The error bars represent the 95% percentile-bootstrap confidence intervals of the RMSE estimates obtained using 10,000 bootstrap resamples.

Fig 12(a) shows the effect of the normalized timing offset on localization accuracy. Under ideal synchronization, the RMSE values are consistent with those reported in Section 3.2. As $\varepsilon_{\tau }$ increases, the RMSE increases at all three SNR levels. For example, at an SNR of 5 dB, the RMSE increases from 8.4 m under ideal synchronization to 11.8 m at $\varepsilon_{\tau }=0.3$. This performance degradation occurs because residual timing offsets introduce frequency-dependent phase errors and reduce the coherence of the compensated signals from different receiving stations.

**Fig 12:**
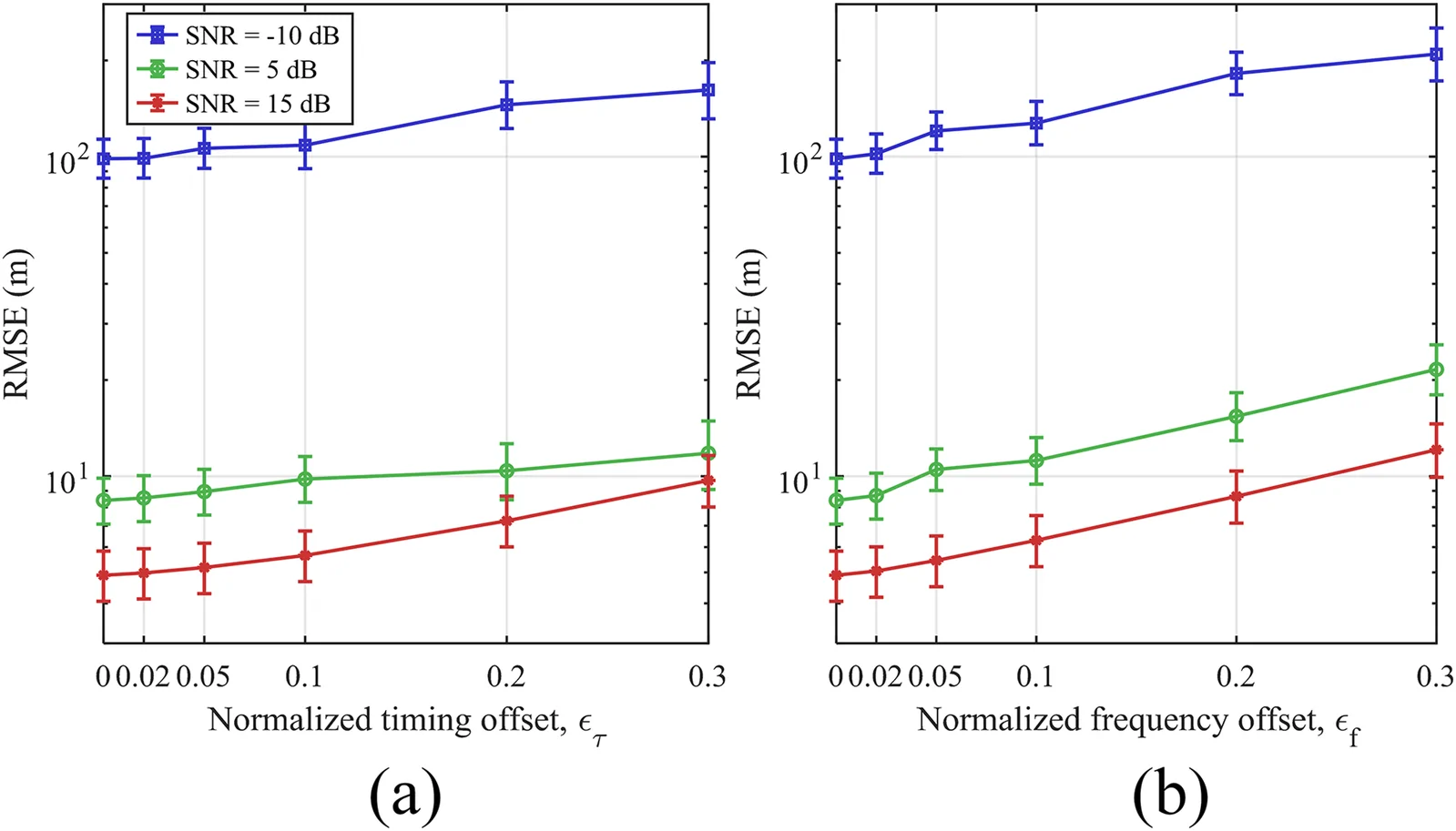
Influence of residual synchronization errors on localization accuracy. (a) RMSE versus normalized timing offset. (b) RMSE versus normalized carrier-frequency offset. Error bars denote the 95% percentile-bootstrap confidence intervals obtained from 500 Monte Carlo trials using 10,000 bootstrap resamples.

Fig 12(b) shows that the carrier-frequency offset causes more pronounced performance degradation. At an SNR of 5 dB, the RMSE reaches 21.6 m when $\varepsilon_{f}=0.3$, which is more than twice the value obtained under ideal synchronization. Similar trends are observed at the other SNR levels. The accumulated phase rotation and spectral leakage caused by the carrier-frequency offset affect target-frequency extraction, frequency-domain covariance-matrix reconstruction, DOA estimation, and subsequent DPD refinement.

For a fixed synchronization-error level, a higher SNR generally results in a lower absolute localization RMSE. Nevertheless, increasing the SNR cannot eliminate the deterministic signal mismatch caused by residual timing and carrier-frequency offsets. Under the normalized error ranges considered in this experiment, the proposed method is more sensitive to carrier-frequency offsets than to timing offsets. The results indicate that the proposed FDCMR-AIR-DPD method maintains relatively stable localization performance under small residual synchronization errors, whereas accurate synchronization calibration remains necessary when the residual offsets become large.

### 3.9 Discussion

The results indicate that the performance improvement of the proposed FDCMR-AIR-DPD method arises from the coordinated operation of its three processing stages. First, FDCMR extracts the selected frequency components and reconstructs the frequency-domain covariance matrix, thereby improving the stability of DOA estimation under low-SNR conditions. Second, the direction-cone intersection exploits the geometric consistency among multiple receiving stations to exclude spatial regions that are inconsistent with the estimated DOAs. Finally, adaptive voxel refinement performs DPD evaluation only within the compressed candidate region. This framework preserves the coherent multi-station information used by conventional DPD while avoiding exhaustive evaluation over the entire three-dimensional search space. The bootstrap confidence intervals obtained from the Monte Carlo simulations further indicate that the observed performance trends are not dominated by finite-sample fluctuations.

The computational advantage of the proposed method should be interpreted in terms of both analytical complexity and the number of DPD objective-function evaluations. For the considered 2000 m × 2000 m × 2000 m search region with a spatial resolution of 5 m, conventional global DPD requires 64,481,201 candidate-position evaluations. With the coverage factor set to i=2 and the maximum number of iterations set to $K_{max}=5$, the proposed adaptive refinement stage evaluates 625 candidate positions. This corresponds to an approximately $1.03\times 10^{\text{5}}$ -fold reduction in the number of DPD objective-function evaluations. This value does not represent an equivalent reduction in the total execution time because FDCMR-DOA estimation, direction-cone construction, cone intersection, and matrix operations still introduce additional computational costs. Nevertheless, the result confirms that the proposed method substantially reduces the dominant spatial-search burden of global DPD. Therefore, i=2 should be regarded as an accuracy-complexity tradeoff under the considered simulation conditions rather than a universally optimal setting.

The deterministic CRB comparison shows that the RMSE of the proposed method follows the theoretical decreasing trend as the SNR increases. The remaining performance gap is mainly associated with voxel discretization, residual DOA estimation errors, and the finite number of adaptive iterations. Therefore, the CRB comparison indicates that the proposed method approaches the theoretical accuracy trend, but it does not imply that the estimator attains the bound. Similarly, the convergence analysis establishes the convergence of the iterative position sequence under the exponentially decreasing resolution rule, but it does not guarantee convergence to the global optimum if the initial candidate region does not contain the true source position. The inter-layer spacing experiment further shows that h/λ provides the best overall performance among the tested configurations by balancing pitch-angle discrimination and phase-ambiguity risk. This value is an empirically favorable configuration for the array geometry considered in this study rather than a universal optimum. In addition, the synchronization-error experiment demonstrates that small residual timing and carrier-frequency offsets cause limited performance degradation, whereas larger offsets noticeably increase the RMSE. The greater sensitivity to carrier-frequency offsets can be attributed to accumulated phase rotation and spectral leakage in the frequency-domain processing stages.

Several limitations should be considered when interpreting these findings. The present study mainly considers a single stationary far-field interference source, known receiving-station coordinates, calibrated double-layer UCAs, and an AWGN-dominated environment. Practical systems may also be affected by multipath propagation, non-line-of-sight components, non-Gaussian noise, receiver-position uncertainty, array-model errors, moving sources, and multiple coherent interference sources. Moreover, the current efficiency analysis is primarily based on asymptotic operation counts and the number of DPD objective-function evaluations rather than implementation-dependent execution time, memory consumption, or hardware measurements. Future work will investigate robust covariance reconstruction under multipath and non-Gaussian conditions, joint estimation and compensation of synchronization errors, localization of moving and multiple sources, and validation using field measurements and hardware implementations. Subject to these limitations, the results support the proposed FDCMR-AIR-DPD method as a practical compromise between localization accuracy, computational efficiency, and low-SNR robustness for three-dimensional interference-source localization.

## 4 Conclusion

To address the problem of three-dimensional radio-frequency interference source localization, this paper proposes an adaptive voxel iterative localization method based on a double-layer UCA and FDCMR-DOA constrained DPD refinement. The FDCMR-DOA estimation results obtained at multiple receiving stations are first used to construct direction cones, and their intersection is employed to determine a compressed candidate region for the interference source. Then, an adaptive voxel iteration strategy is applied within this compressed region to refine the source position with controllable computational cost.

The proposed method has three main advantages. First, the double-layer UCA configuration improves pitch-angle discrimination by exploiting the vertical phase difference between the two array layers. Second, the FDCMR-DOA based cone-intersection strategy compresses the original three-dimensional global search region before DPD evaluation, thereby avoiding exhaustive DPD search over the entire positioning space. Third, the adaptive voxel refinement strategy further improves localization accuracy while keeping the number of DPD evaluations limited.

Theoretical analysis and simulation results demonstrate the effectiveness of the proposed method. Under the simulation setting with a 2000 m × 2000 m × 2000 m search region and a global DPD resolution of 5 m, the number of DPD evaluations is reduced from 64,481,201–625, corresponding to a reduction of approximately $1.03\times 10^{\text{5}}$ times. The proposed method achieves an RMSE of 153.2 m at −15 dB, which is about 73.3% lower than that of the fixed-grid DPD method. Additional simulations with confidence intervals, convergence verification, CRB comparison, and inter-layer spacing analysis further confirm the robustness and efficiency of the proposed method. Overall, the proposed FDCMR-AIR-DPD method provides a practical balance between localization accuracy and computational complexity for three-dimensional interference source localization under low-SNR conditions.
